# A novel canine kidney cell line model for the evaluation of neoplastic development: karyotype evolution associated with spontaneous immortalization and tumorigenicity

**DOI:** 10.1007/s10577-015-9474-8

**Published:** 2015-05-10

**Authors:** R. Omeir, R. Thomas, B. Teferedegne, C. Williams, G. Foseh, J. Macauley, L. Brinster, J. Beren, K. Peden, M. Breen, A. M. Lewis

**Affiliations:** Laboratory of DNA Viruses, Division of Viral Products, Office of Vaccines Research and Review, Center for Biologics Evaluation and Research, Food and Drug Administration, 10903 New Hampshire Ave, Silver Spring, MD 20993 USA; Department of Molecular Biomedical Sciences, College of Veterinary Medicine, North Carolina State University, 1060 William Moore Drive, Raleigh, NC 27607 USA; Center for Comparative Medicine and Translational Research, North Carolina State University, Raleigh, NC 27607 USA; Division of Veterinary Resources, National Institutes of Health, Bethesda, MD 20892 USA; Office of Counter-Terrorism and Emergency Coordination, Center for Drug Evaluation and Research, Food and Drug Administration, Silver Spring, MD 20993 USA; Cancer Genetics Program, University of North Carolina Lineberger Comprehensive Cancer Center, Chapel Hill, NC 27599 USA; Center for Human Health and the Environment, North Carolina State University, Raleigh, NC 27607 USA

**Keywords:** Neoplastic transformation, Tumorigenicity, Canine chromosomes, Comparative genomic hybridization (CGH), Fluorescence in situ hybridization (FISH), Madin-Darby canine kidney (MDCK) cell line, CKB1-3T7 cell line

## Abstract

The molecular mechanisms underlying spontaneous neoplastic transformation in cultured mammalian cells remain poorly understood, confounding recognition of parallels with the biology of naturally occurring cancer. The broad use of tumorigenic canine cell lines as research tools, coupled with the accumulation of cytogenomic data from naturally occurring canine cancers, makes the domestic dog an ideal system in which to investigate these relationships. We developed a canine kidney cell line, CKB1-3T7, which allows prospective examination of the onset of spontaneous immortalization and tumorigenicity. We documented the accumulation of cytogenomic aberrations in CKB1-3T7 over 24 months in continuous culture. The majority of aberrations emerged in parallel with key phenotypic changes in cell morphology, growth kinetics, and tumor incidence and latency. Focal deletion of *CDKN2A*/*B* emerged first, preceding the onset and progression of tumorigenic potential, and progressed to a homozygous deletion across the cell population during extended culture. Interestingly, CKB1-3T7 demonstrated a tumorigenic phenotype in vivo prior to exhibiting loss of contact inhibition in vitro. We also performed the first genome-wide characterization of the canine tumorigenic cell line MDCK, which also exhibited *CDKN2A*/*B* deletion. MDCK and CKB1-3T7 cells shared several additional aberrations that we have reported previously as being highly recurrent in spontaneous canine cancers, many of which, as with *CDKN2A*/*B* deletion, are evolutionarily conserved in their human counterparts. The conservation of these molecular events across multiple species, in vitro and in vivo, despite their contrasting karyotypic architecture, is a powerful indicator of a common mechanism underlying emerging neoplastic activity. Through integrated cytogenomic and phenotypic characterization of serial passages of CKB1-3T7 from initiation to development of a tumorigenic phenotype, we present a robust and readily accessible model (to be made available through the American Type Culture Collection) of spontaneous neoplastic transformation that overcomes many of the limitations of earlier studies.

**Electronic supplementary material:**

The online version of this article (doi:10.1007/s10577-015-9474-8) contains supplementary material, which is available to authorized users.

## Introduction

It has been evident since the 1940s that untreated mammalian cells propagated in culture can evolve during serial passage to express a tumorigenic phenotype, defined as the capacity to form tumors when inoculated into immunocompromised rodents (Gey 1941; Earle et al. 1943). Since these early observations, several immortalized cell lines, established from a variety of different species, have been documented to develop the ability to form tumors in vivo, spontaneously and without chemical or genetic modification (Macpherson and Stoker 1962; Defendi et al. 1963; Aaronson and Todaro 1968; Jarrett and Macpherson 1968; Contreras et al. 1985; Kraemer et al. 1987; Rubin et al. 1990; United States of America Food and Drug Administration 2005; Manohar et al. 2008; Liu et al. 2010; Onions et al. 2010; Omeir et al. 2011). Notable among these are the Madin-Darby canine kidney (MDCK) cell line (Madin and Darby 1958; United States of America Food and Drug Administration 2005; Gregersen 2008; Liu et al. 2010; Onions et al. 2010; Omeir et al. 2011; Brinster et al. 2013) and the VERO line of African green monkey kidney (AGMK) cells (Contreras et al. 1985; Manohar et al. 2008). The MDCK and VERO cell lines are used as substrates for viral vaccine production (Barrett 1980; Montagnon 1989; Medema et al. 2006; Betakova et al. 2013). The use of tumorigenic cell lines in this context has raised concerns over the potential for vaccine-mediated transfer of neoplastic activity by cellular components, such as oncogenic viruses and cellular DNA (Sheng-Fowler et al. 2009), and subcellular components such as exosomes and microRNAs (Fevrier and Raposo 2004; Lujambio and Lowe 2012). A rigorous assessment of these concerns requires a comprehensive understanding of the complex processes that govern the acquisition of a tumorigenic phenotype. Studies in vitro have revealed a multistage series of cellular events evolving from genomic and epigenetic alterations that provide growth advantages to replicating cells (Barrett 1980; Thomassen 1986; Rubin 2005; Buonanno et al. 2011). These investigations have focused heavily on individual loci of specific interest, and the few genome-wide reports have been limited in resolution, confounding the ability to correlate molecular alterations with biological behavior. Furthermore, many prior studies represent retrospective investigations of induced rather than spontaneous transformation, often in rodent cell line models, restricting opportunities for correlation of in vitro observations with in vivo data from naturally occurring tumors within the same species.

Our recent evaluation of the complex tumorigenic phenotype of MDCK cells (Omeir et al. 2011; Brinster et al. 2013) offers the potential for molecular characterization of this widely used cell line in context with the expanding catalog of genomic profiling data arising from naturally occurring tumors of domestic dogs (Breen 2009; Thomas et al. 2009, 2011, 2014; Angstadt et al. 2011; Hedan et al. 2011; Poorman et al. 2015; Shapiro et al. 2015). Unfortunately, the biological characteristics of the MDCK cell line following initiation of the original culture are not well documented (Madin and Darby 1958; Gaush et al. 1966; Leighton et al. 1969; Rindler et al. 1979; Saier 1981), and the event that resulted in transformation of MDCK cells into a continuous cell line is not recorded. Furthermore, several biologically heterogeneous derivatives of the original line are in use today (Dukes et al. 2011). These limitations, coupled with the fact that early nontumorigenic MDCK cell populations are no longer available, preclude the ability to investigate the evolution of this phenotype in a progressive manner.

We describe the development of a new canine kidney cell line, CKB1-3T7, which permits a prospective investigation of emerging neoplastic events in vitro and overcomes many of the limitations of earlier studies. We present a comprehensive cellular and molecular characterization of serial passages of the CKB1-3T7 cell line banked at regular intervals over a 2-year period of continuous culture. These cell banks encompass the spectrum of events leading from cell line initiation to immortalization and the development and progression of tumorigenic activity. We compare these data with biological and cytogenomic profiles we obtained from the MDCK cell line to identify conserved features that support the independent association of specific genomic alterations with cellular phenotype. Finally, we discuss the degree to which these observations recapitulate the profiles of genomic instability evident in naturally occurring cancers. We propose that this series of well-characterized reagents confers a robust and widely accessible system for systematically elucidating the biological intricacies of neoplastic development in mammals.

## Materials and methods

### Development of the CKB1-3T7 cell line

Healthy canine kidney tissue was provided by the National Institutes of Health (NIH) Division of Veterinary Services. Kidneys were obtained from a healthy 2-year-old male Beagle (Charles River Laboratories, Frederick, MD) used in an Institutional Animal Care and Use Committee (IACUC)-approved study of the effects of intra-aortic balloon counterpulsation on septic shock (Solomon et al. 2009). Histopathological evaluation of formalin-fixed tissue sections from the specimen was unremarkable. Representative fresh tissue was washed three times with phosphate-buffered saline (PBS), minced into 2–3 mm pieces, and disaggregated with 0.25 % trypsin (Mediatech, Manassas, VA) for 1 h at 37 °C with shaking. The disaggregated cells were washed three times with PBS and resuspended in 15 mL Dulbecco’s modified Eagle’s medium (DMEM, Mediatech) supplemented with 10 % fetal bovine serum (FBS, Lot ASL31024; Hyclone, Logan, UT), 2 mM l-glutamine (Mediatech) (DMEM-10), and antibiotics [penicillin 100 U/mL, streptomycin 100 μg/mL, gentamicin 50 μg/mL (Mediatech), and 10 μg/mL tetracycline (Webster Veterinary Supply, Devens, MA)]. Cells were plated into two T75 flasks (Corning Incorporated, Corning, NY) and incubated at 37 °C, 5 % CO_2_. Beginning at passage (p) 3, cells were cultured in DMEM-10 without antibiotics. Upon reaching confluence, approximately 4 × 10^5^ cells/mL were cryopreserved by resuspension in 1 mL of 7.5 % dimethylsulfoxide (DMSO, Sigma Aldrich, St. Louis, MO) in DMEM-10 and stored in the vapor phase of liquid nitrogen. For cell line establishment, p3 cells (approximately 4 × 10^5^ cells) were thawed, plated in a T25 flask (Corning) in DMEM-10, and incubated at 37 °C, 5 % CO_2_. The medium was replaced every 3–4 days for 6 weeks by which time the cells were confluent. By p9, cell growth was such that cells plated at 3 × 10^5^ cells/T25 flask were consistently 95–98 % confluent after 7 days in culture, making it possible to initiate serial passaging.

For serial passage (p10–p90), at 7-day intervals, cells from two T25 flasks were washed with PBS-EDTA (Teknova, Hollister, CA), detached by incubation in 0.25 % trypsin (Mediatech) and 0.53 mM EDTA (Quality Biological Inc., Gaithersburg, MD), and pooled. The number of cells was determined using a Cellometer (Nexcelom Bioscience, Lawrence, MA), and the cell suspension was used to seed T25 flasks with 3 × 10^5^ cells (1.2 × 10^4^ cells/cm^2^) in DMEM-10 for the next passage. During serial passage, cell monolayers, cell morphology, and cell-sheet confluence were monitored daily. Based on the origin of the tissue and the method of culture (Todaro and Green 1963), the cell line was designated CKB1-3T7: canine kidney Beagle1 [CKB1]—3 × 10^5^ cells, transferred [*T*] every *7* days. The same lot of FBS was used throughout the establishment and propagation of CKB1-3T7 cells. The cell line tested negative for 31 rodent agents (26 viruses and mycoplasma species/pulmonis; IDEXX RADIL, Columbia, MO). Cells banked every ten passages from p10 to p90 will be available from the American Type Culture Collection (ATCC, Manassas, VA).

### Cell growth characteristics

The doubling time (DT) of CKB1-3T7 cells in hours was determined at each passage. Every 7 days, cells from two of the T25 flasks plated at 3 × 10^5^ cells/flask for passage were harvested. The number of cells plated at passage initiation and the number of cells after 7 days (168 h) in culture were used to determine the doubling time using the formula: 1 / [((log *B* − log *A*) × 3.32) / 168], where *A* = initial number of cells plated, *B* = final number of cells in the flask, and 3.32 represents 1/log_2_. Population doubling levels (PDL) were determined by the formula: PDL = PDL_i_ + 3.32 × (total viable cells at harvest/total viable cells at plating), where PDL_i_ = the PDL of the previous passage.

To evaluate the cell growth characteristics at ten-passage intervals, a 95–98 % confluent monolayer of CKB1-3T7 cells from a T150 flask seeded at 1.2 × 10^4^ cells/cm^2^ was split into 30 T25 flasks at 3 × 10^5^ cells/flask and the medium replaced every 3–4 days for 20 days (480 h) to 28 days (672 h). Every 2–3 days, cells in two T25 flasks were trypsinized and pooled, and the average number of cells/flask was determined. Assays were performed in duplicate, and the values for the average numbers of cells were used to graph population growth over time.

### Cell migration and tumorigenicity assays

Wound-healing assays were undertaken to evaluate potential changes in the migration phenotype of CKB1-3T7 cells at different passage levels. One million cells were plated in triplicate in 60-mm-diameter culture dishes. When cultures reached 90 % confluence, cells were serum starved for 8 h, and the monolayers were wounded with a P200 pipette tip, washed with PBS, and cultured in DMEM-10. Phase-contrast images of cell migration into the wounded area were photographed at 0, 12, 24, and 36 h using an Olympus IX51 microscope with a DP72 camera and a ×20 objective. Cell migration (% of wound closure) was determined at 36 h by the formula [(initial wound size − wound size at time of measurement) / initial wound size] × 100.

Tumorigenicity studies were performed as described previously (Omeir et al. 2011) in both newborn and adult mice due to the difference in sensitivity (newborn mice being more sensitive) to tumor formation. Briefly, newborn (<72 h old) and adult (4–6 weeks old) athymic nude mice (Frederick Cancer Research Facility, National Cancer Institute, NIH) were inoculated subcutaneously in the dorsal region of the thorax above the scapulae with 10^7^ cells in 0.1 mL PBS per mouse. The animals were examined weekly for 12 months for the presence and progression of tumors. Progressive tumor growth was determined by two-dimensional measurements at weekly intervals using a VWR Digital Caliper (VWR International, Radnor, PA). Tumor incidence data, represented by the percent of tumor-free animals, were plotted as Kaplan-Meier survival curves. Mice were euthanized when tumors reached approximately 20 mm in any dimension. All institutional and national guidelines for the care and use of laboratory animals were followed, and the protocols for these assays were approved by the IACUC of the Center for Biologics Evaluation and Research.

### Preparation of selected cell line passages for cytogenomic analysis

Cytogenomic analysis of CKB1-3T7 cells was performed initially at p7, p15, and p22 and subsequently at intervals of approximately ten passages (p32, p43, p52, p62, p73, and p92), representing a period of continual propagation over 24 months. Duplicate flasks for each selected passage were allowed to approach confluence, the cells were rinsed with Hanks’ balanced salt solution (Mediatech), disaggregated with 0.05 % trypsin/EDTA (Mediatech), pooled, and split equally into three new T75 flasks in 25 mL of DMEM-10 per flask. Cultures were propagated until approaching confluence, at which time the cells from one flask were cryopreserved in 10 % DMSO/90 % FBS (Mediatech). Cells from the two remaining flasks were arrested at metaphase by exposure to 50 ng/mL Karyomax (Gibco/Life Technologies, Grand Island, NY) for 16 h (flask 1) and 100 ng/mL Karyomax for 4 h (flask 2). Cells from both flasks were recovered by trypsin-EDTA treatment and pooled. The combined cell number was determined using a Cellometer and divided into two equivalent aliquots. The first aliquot was harvested with conventional hypotonic treatment and 3:1 methanol/glacial acetic acid fixation. Metaphase chromosome preparations were dropped onto clean, uncharged glass microscope slides; cured for 3–5 days at room temperature; dehydrated through 70, 90, and 100 % ethanol baths; and stored at −80 °C until required. The second aliquot of cells was rinsed in PBS and used for isolation of total genomic DNA (Qiagen DNeasy Blood and Tissue Kit, Qiagen, Valencia, CA).

### Genomic DNA copy number profiling analysis of CKB1-3T7 cells

Oligonucleotide-array comparative genomic hybridization (oaCGH) analysis was performed as described previously (Poorman et al. 2015; Thomas et al. 2014; Shapiro et al. 2015), using a custom 1,000,000-feature microarray (Agilent Technologies, Santa Clara, CA). The array comprises repeat-masked ∼60-mer oligonucleotides distributed at approximately 2.4 kb intervals throughout the domestic dog genome sequence assembly (Lindblad-Toh et al. 2005). The genome-wide DNA copy number profile of each of the nine serial cell line passages (“test” samples) was evaluated in a series of pairwise hybridizations against a common reference sample comprising equimolar quantities of blood-derived DNA acquired from five, young (<3 years), clinically healthy, male Beagles during routine wellness examinations. Array image data were processed using Feature Extraction version 10.10 and Genomic Workbench version 7 (Agilent Technologies). Raw data were filtered to exclude probes exhibiting nonuniform hybridization or signal saturation. Nonrandom, recurrent DNA copy number aberrations (CNAs) were defined using the ADM2 segmentation algorithm in Genomic Workbench with a threshold of six, based on a minimum of three consecutive probes with log_2_ test:reference values ≥0.2 (copy number gain) or ≤−0.2 (copy number loss), resulting in an effective resolution of ∼4.8 kb (two intervals of ∼2.4 kb). High-amplitude gains and losses were defined using default log_2_ test:reference values of >1.14 and <−1.1, respectively. Genes and uncharacterized coding sequences within recurrent CNAs were defined using the UCSC canine genome sequence browser (http://genome.ucsc.edu/). Discrete genomic regions are herein denoted according to their cytogenetic location and their megabase (Mb) coordinates within the dog genome sequence assembly (Lindblad-Toh et al. 2005). Dog chromosome Y (*Canis familiaris* (CFA) Y) was excluded from analysis due to the absence of this chromosome in the female dog genome sequence assembly.

### Targeted fluorescence in situ hybridization (FISH) analysis of CKB1-3T7

Key CNAs identified by oaCGH analysis were selected for targeted FISH analysis based on their emergence and/or change in amplitude during cell line propagation. FISH analysis was performed using metaphase chromosome preparations obtained from an aliquot of the same cell population of each passage from which DNA was extracted for use in oaCGH. Two types of FISH probes were used. Fluorescently labeled single-locus probes (SLPs) were generated using cytogenetically validated bacterial artificial chromosome (BAC) clones from the RPCI-81 or CHORI-82 canine BAC libraries (https://bacpac.chori.org) (Thomas et al. 2007, 2008). Whole chromosome paint probes were prepared from bivariate flow-sorted dog chromosomes (Breen et al. 1999b). All FISH probes were first hybridized to metaphase chromosome preparations from clinically healthy dogs to validate signal quality and location (Breen et al. 1999b; Thomas et al. 2008). Pools of differentially labeled FISH probes were then used to assess the range and distribution of both structural and numerical chromosome aberrations in up to 50 cells from each of the passages evaluated by oaCGH analysis.

### Cytogenomic evaluation of the MDCK cell line

Cells from three different lots of parental MDCK cells (NBL-2, catalog number CCL-34) were obtained from the ATCC. The biological characteristics of these cells have been described previously (Omeir et al. 2011). Briefly, MDCK vial 1 (V1: lot no. 3563161, frozen January 30, 2004) was acquired at p55 and maintained in DMEM-10. MDCK vial 2 (V2: lot no. 4398972, frozen January 20, 2006) and vial 3 (V3: lot no. 7643577, frozen July 20, 2007) were received at p56 and p55, respectively, and were propagated using ATCC-formulated EMEM supplemented with 10 % FBS. V1 and V2 cells originated from the same token lot established at p52. V3 cells were established from a different token lot that was also established at p52. These different MDCK cell lots exhibit differing tumor-forming capacity in athymic nude mice, with log_10_ tumor-producing doses at 50 % endpoints [TPD_50_] of 5.2 (V1), 4.4 (V2), and 5.8 (V3) (Omeir et al. 2011). Cells from all three lots were evaluated by oaCGH and FISH analyses as described above.

## Results

### Evolution of growth rate, morphology, and tumorigenicity in CKB1-3T7 cells

**Fig. 1:**
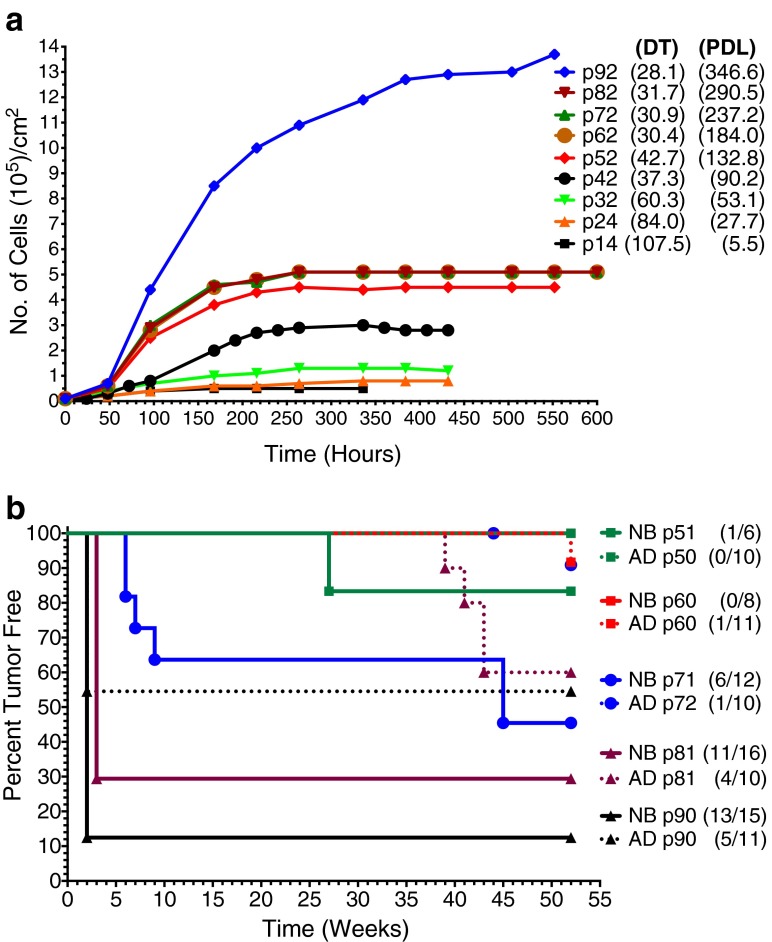
CKB1-3T7 cell growth characteristics and tumorigenicity. **a** Population growth, cell doubling times (*DT*), and population doubling levels (*PDL*) of CKB1-3T7 cells sampled at approximately ten passage intervals from cell line initiation. **b** Kaplan-Meier curves of tumor formation in newborn (*NB*) and adult (*AD*) athymic nude mice after inoculation with 10^7^ CKB1-3T7 cells. The *graphs* plot the percent of mice that are tumor-free over time, while the *numbers in parentheses* indicate the tumor incidence at end of the experiment

During the first 30 passages after initiation, CKB1-3T7 cells demonstrated a gradual increase in growth rate, with the DT decreasing 1.8-fold from 107.5 h at p14 to 60.3 h at p32 (Fig. 1a). During this period, the cell population exhibited a mixture of cells with epithelial and mesenchymal characteristics (SOM Fig. 1A). By p42, the DT decreased further to 37.3 h (Fig. 1a), and the morphological composition of the cell culture began to evolve, becoming predominantly cuboidal by p50 (SOM Fig. 1B). Both cell morphology and the DT subsequently remained stable for the next 20 passages (30.4 h at p62, 30.9 h at p72, and 31.7 h at p82). Loss of contact inhibition emerged by p90, with the cell sheets exhibiting multilayer areas throughout the flask (SOM Fig. 1C), resulting in a decrease in DT, an increase in cell density, and a sustained population expansion (Fig. 1a).

**Fig. 2:**
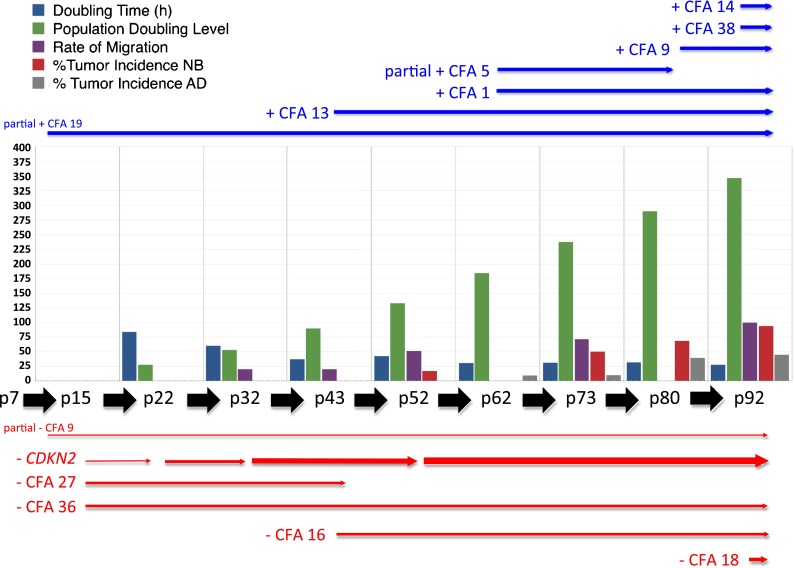
Integrated summary of the evolution in cellular and cytogenomic characteristics during long-term propagation of CKB1-3T7 cells. CNAs identified by oaCGH analysis are shown against the interval in which they emerged, denoted in *blue* (gain) or *red* (loss). The change in breadth of the *arrow* denoting the *CDKN2A*/*B* deletion reflects the progressive increase in the amplitude of this CNA in the successive passages analyzed. Overlaid on these data are key time points associated with the biological phenotype of each passage

Wound-healing and tumor formation assays were performed to assess the relationship between cell migration and tumor-forming ability in CKB1-3T7 cells. At p32 and p42, there was little or no motility observed during wound-healing assays at 36 h, but there was a progressive increase in migratory capacity from p52 (51.4 % wound closure) to p74 (71.4 % wound closure), with the wound being 100 % closed at p94 (SOM Fig. 2). From p50, CKB1-3T7 cells showed a gradual onset of tumor-forming capacity and a decrease in the interval between cellular inoculation and the appearance of a tumor (tumor latency). The incidence of tumor formation in newborn athymic nude mice increased from 1/6 (83 % tumor-free) at p51 to 13/15 (13 % tumor-free) at p90, with tumor latency falling from 26–28 weeks (p51) to 2–3 weeks (p90) (Fig. 1b). In contrast, tumor formation in adult nude mice was first observed at p60 (1/11, 91 % tumor-free), increasing to 5/11 (55 % tumor-free) at p90, with a marked decrease in tumor latency from 52–53 weeks (p60) to 2–3 weeks (p90) (Fig. 1b). Two tumors that formed in newborn athymic nude mice inoculated with p90 cells regressed.

### Evolution of genomic profiles during propagation of CKB1-3T7 cells

**Fig. 3:**
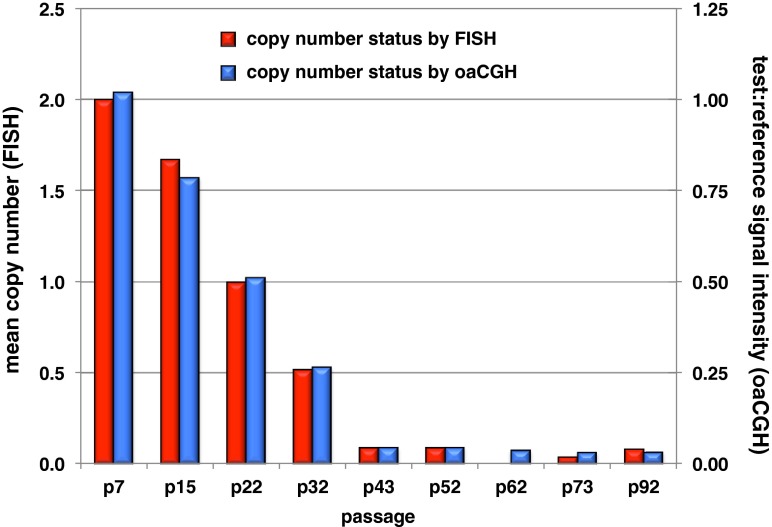
Assessment of DNA copy number status at the *CDKN2A*/*B* locus on CFA 11q16 in CKB1-3T7 cells. The mean copy number of the region as determined by FISH analysis of serial passages of CKB1-3T7 cells is shown on the *primary vertical axis to the left*. The *secondary vertical axis on the right* indicates the corresponding test:reference signal intensity value at this locus as determined by oaCGH analysis. These values are plotted on a linear (rather than logarithmic scale) to aid comparison with FISH data for the same passage

**Fig. 4:**
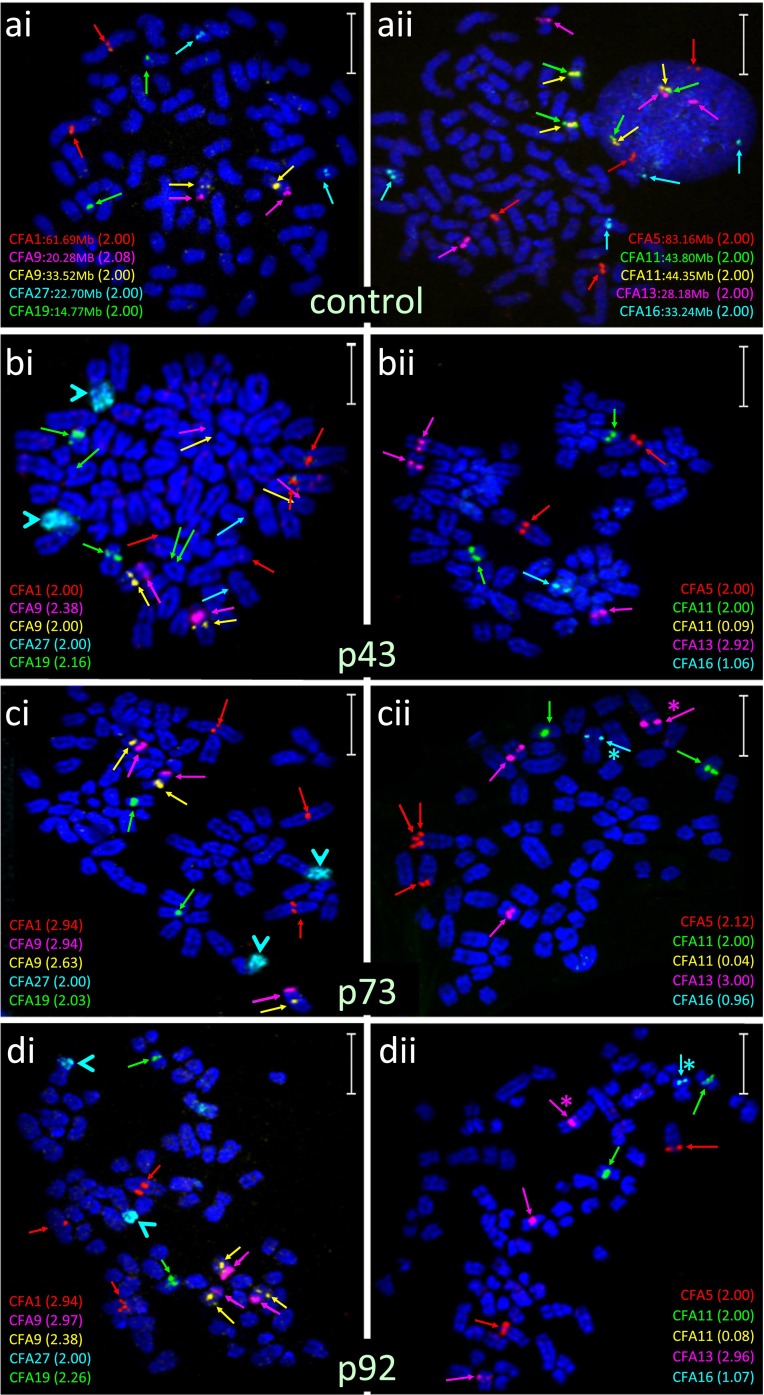

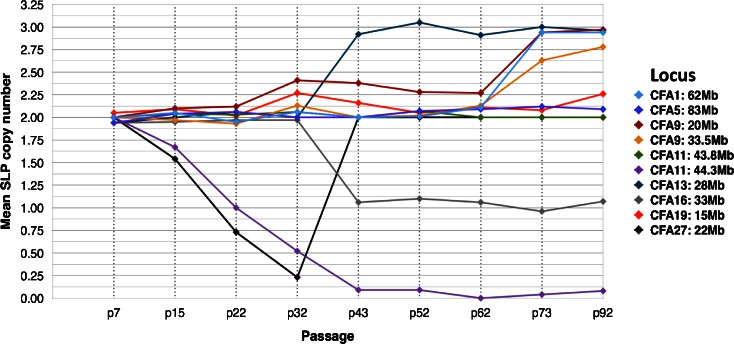
Multicolor FISH analysis of CKB1-3T7 cells over successive passages. **a** FISH analysis with two five-color probe sets. SLPs were targeted to detect the central region of the chromosome or subchromosomal region of interest. *Panel 1* (*ai*, *bi*, *ci*, and *di*) comprises SLPs for regions of CFA 1:62 Mb (*red*), CFA 9:20 Mb (*magenta*), CFA 9:33.5 Mb (*gold*), CFA 19:15 Mb (*green*), and CFA 27:22 Mb (*aqua*), and *panel 2* (*aii*, *bii*, *cii*, and *dii*) comprises SLPs for regions of CFA 5:83 Mb (*red*), CFA 11:43.8 Mb (*green*), CFA 11[*CDKN2A/B*]:44.3 Mb (*gold*), CFA 13:28 Mb (*magenta*), and CFA 16:33 Mb (*aqua*). The BAC clone representing CFA 13 also contains the full length of the *MYC* gene (Thomas et al. 2007, 2008). The *numbers in parentheses* adjacent to the probe in each figure refer to the mean SLP copy number derived from counts of up to 50 cells from clinically normal control dog chromosomes (*ai*, *aii*) and from CKB1-3T7 cell line chromosomes at p43 (*bi*, *bii*), p73 (*ci*, *cii*), and p92 (*di*, *dii*). *Aqua arrowheads* in *bi*, *ci*, and *di* indicate use of a CFA 27 whole chromosome paint probe in place of the SLP to permit structural as well as numerical analysis. *Pink* and *aqua asterisks* in *cii* and *dii* indicate abnormal derivatives containing at least a segment of CFA 13 (*pink asterisk*) and CFA 16 (*aqua asterisk*). BAC addresses for clones used to generate each SLP are provided in SOM Table 1. *Size bars* represent 5 μm. **b** Graphical representation of the mean copy number of each SLP assessed across all CKB1-3T7 cell passages analyzed. The SLP at CFA 11:44.3 Mb includes the *CDKN2A*/*B* locus

Genome-wide DNA copy number profiling demonstrated that the evolution in cell growth, morphology, and tumor-forming potential of CKB1-3T7 cells during propagation from p7 to p92 was accompanied by a progressive accumulation of DNA copy number aberrations (Fig. 2 and SOM Fig. 3). Cells at p7 exhibited a grossly balanced DNA copy number profile in oaCGH analysis, and the number and morphology of chromosomes was consistent with that of the normal dog karyotype [2*n* = 78, (Breen et al. 1999a)]. Apparent regional genomic imbalances at p7, including copy number losses of CFA 9q14:20.7 Mb and CFA 19q13.1:14.4 Mb, coincided with natural DNA copy number polymorphisms that have been described in the domestic dog (Chen et al. 2009; Nicholas et al. 2009) and remained evident in subsequent passages. oaCGH analysis of cells at p15 detected deletions of CFA 27 and CFA 36 and also a low amplitude focal deletion of a 1.04 Mb interval on CFA 11q16:44.17–45.20 Mb, a region containing the full length of the *MTAP*, *CDKN2A*/*B*, and *DMRTA1* loci. Targeted FISH analysis demonstrated hemizygous interstitial deletion of this interval on CFA 11q16 in 49 % of cells evaluated at p15 (Figs. 3 and 4). The amplitude (log_2_ test:reference ratio) of this deletion continued to increase progressively in p22 and p32 cells (Figs. 3 and 4), at which time genomic profiles remained otherwise consistent with that of cells at p15. At p32, FISH analysis demonstrated hemizygous deletion of CFA 11q16:44.17–45.20 Mb in 50 % of cells, with the remainder of the population exhibiting homozygous deletion. The amplitude of the aberration began to plateau at p43, at which time this region of CFA 11q16 was undetectable in 91.5 % of cells by FISH analysis (Figs. 3 and 4). oaCGH analysis of p43 cells also revealed that the copy number status of CFA 27, which was reduced to *n* = 1 in 24.1 % of cells at p32, had returned to a balanced state. FISH analysis using a chromosome paint probe representing CFA 27 showed that this return to *n* = 2 coincided with the appearance of two derivative chromosomes, each formed by fusion of one copy of CFA 27 with a second, unidentified chromosome (Fig. 4). These derivatives remained evident throughout continued propagation of CKB1-3T7. oaCGH analysis at p43 also revealed gain of CFA 13 and loss of CFA 16, which FISH analysis showed to be consistent with trisomy and monosomy, respectively (Fig. 4). No additional changes were evident at p52. At p62, gain of CFA 1 became evident along with a low amplitude gain of ∼9.6 Mb at CFA 5q34-q35:76.21–85.77 Mb, followed at p73 by gain of CFA 9 and an increase in the amplitude of gain at CFA 5q34-q35. The discrete gain of CFA 5q34-q35:76.21–85.77 Mb was evident in FISH analysis as a tandem duplication in 23 % of p73 cells evaluated (Fig. 4). By p92, this aberration was detected in only 3.1 % of cells, consistent with return to a grossly balanced state indicated by oaCGH analysis. By p92, CKB1-3T7 cells had also accumulated gain of CFA 14 and 38 and deletion of the central region of CFA 18 (CFA 18q12-q24:13.81–43.74 Mb). The amplitude of the interstitial deletion of CFA 11q16:44.17–45.20 Mb in oaCGH analysis reached a maximum at p92, at which time this region was rarely detectable in cells analyzed by FISH analysis (Figs. 3 and 4). The genomic location of all FISH probes is provided in SOM Table 1.

### Genomic profiling analysis of MDCK cells

**Fig. 5:**
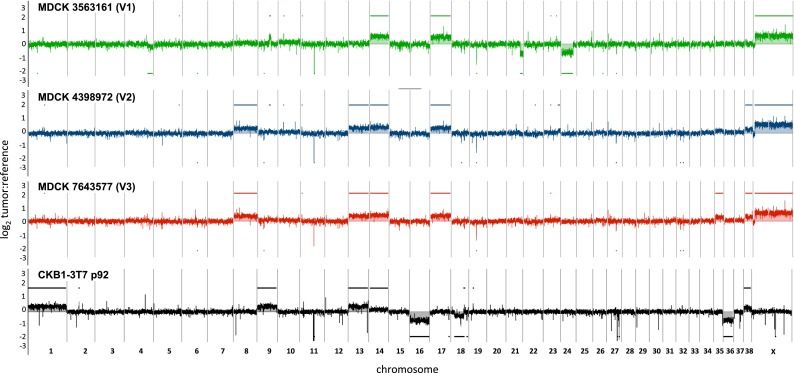
DNA copy number profiles of MDCK V1, MDCK V2, and MDCK V3 cells obtained by oaCGH analysis. The *horizontal axis* indicates the genomic location of arrayed probes and the *vertical axis* indicates the log_2_ test:reference value at that locus for each of the three MDCK lots. *Color-matched horizontal bars* above and below each CGH profile delineate regions of DNA copy number gain and loss, respectively. Each MDCK profile shows an apparent copy number increase of CFA X, relative to the male reference. Data from CKB1-3T7 cells at p92 are included to aid comparison with data from MDCK cells

**Fig. 6:**
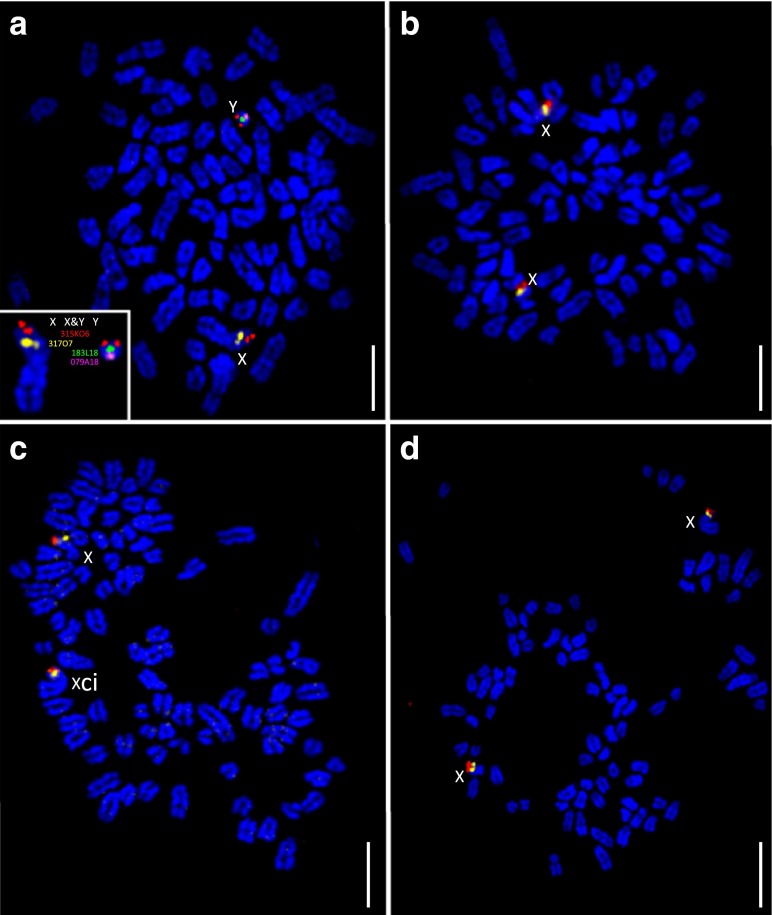
Multicolor FISH of MDCK cells using sex chromosome-specific SLPs. SLPs were generated using clones from the CHORI-82 canine BAC library [CFA Xp21 (317O17, *gold*) and Xp22.3/Yp11.3 (315K06, *red*)] and the RPCI-81 canine BAC library [CFA Yq11.1 (079A18, *pink*) and Yp11.1 (183L18, *green*)]. **a** Hybridization of each SLP to clinically healthy male dog control chromosomes. The *inset* shows enlarged and correctly oriented CFA X and CFA Y from this metaphase chromosome spread with each SLP signal identified by the corresponding BAC clone address. Clone 315K06 hybridized to both CFA X and Y, consistent with its location within the canine pseudoautosomal region. The same SLP panel was hybridized to **b** MDCK V1 cells, **c** MDCK V2 cells, and **d** MDCK V3 cells. *Size bars* represent 5 μm

**Fig. 7:**
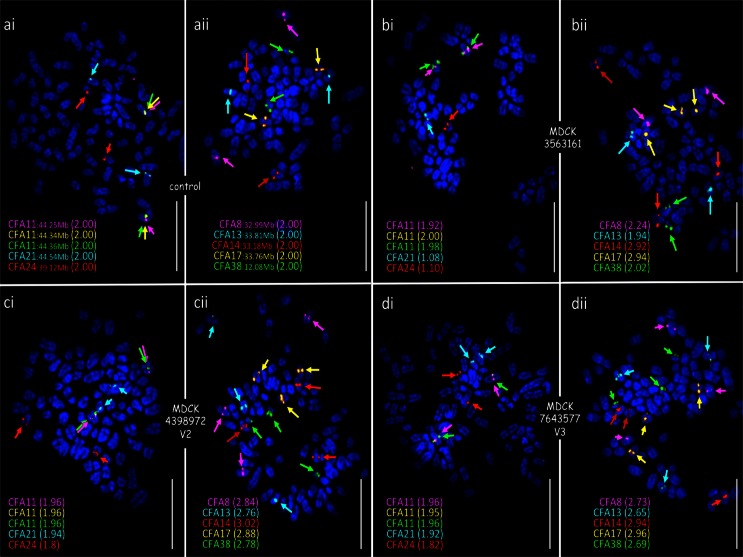
Targeted FISH analysis of MDCK cells. Metaphase chromosome preparations from *a* a clinically healthy control dog, *b* MDCK V1 cells, *c* MDCK V2 cells, and *d* MDCK V3 cells were assessed using two SLP panels targeting loci on CFA 11, 21, and 24 (*panel i*) and CFA 8, 13, 14, 17, and 38 (*panel ii*). For each panel, the genomic location of the center of the BAC clone used is indicated in the control cells (*ai* and *aii*). The *numbers in parentheses* adjacent to each chromosome ID are the mean copy number of that probe obtained from enumeration of SLP signals in up to 50 cells. BAC addresses for clones used to generate each SLP are provided in SOM Table 1

oaCGH analysis of MDCK V1, V2, and V3 cells demonstrated relatively limited DNA copy number imbalance (Fig. 5). This was supported by observations from chromosome enumeration (V1 mean = 77, range 76–79; V2 and V3 mean = 80, range 78–82 and 78–83, respectively). Cells from all three lots showed elevated copy number along the length of CFA X relative to the male reference in oaCGH analysis (Fig. 5) and exhibited two bi-armed (submetacentric) chromosomes, both of which hybridized CFA X-specific FISH probes (Fig. 6). All other chromosomes appeared to be acrocentric. No CFA Y signal was detected in FISH analysis (Fig. 6), and each cell lot tested negative for the CFA Y-specific sex-determining region (SRY) using PCR analysis ((Bannasch et al. 2005; data not shown). The natural DNA copy number polymorphisms observed for CFA 9q14:20.7 Mb and CFA 19q13.1:14.4 Mb in CKB1-3T7 cells were also evident in cells from all three lots of MDCK cells. All three showed targeted interstitial deletion of a 60-kb region at CFA 11q16:44.23–44.29 Mb, consistent with homozygous loss of *CDKN2A*/*B* and the distal end (exons 7 and 8) of the *MTAP* gene, and also shared gain along the length of CFA 14 and CFA 17. In contrast to the CKB1-3T7 cell line, FISH analysis of this region in MDCK cells showed a near-balanced copy number status (*n* ≥ 1.96) in all cells scored (Fig. 7). This was consistent with the smaller interval of deletion around the *CDKN2A*/*B* region in MDCK cells (60 kb) versus CKB1-3T7 cells (1.04 Mb), relative to the size of the BAC clone used in FISH analysis of that region (172 kb).

Other DNA copy number aberrations were evident in oaCGH profiles of only one or two of the MDCK lots. V2 and V3 cells shared common gains of CFA 8, CFA 13, and CFA 38. V3 cells showed gain of CFA 35, which was also evident at low amplitude in V2 cells but fell outside the threshold required for classification as aberrant in oaCGH. V1 cells showed several aberrations that were not evident in the other two cell lots, including partial losses of CFA 4q33-qtel:73.67–91.48 Mb, CFA 21q24.2-qtel:44.37–53.93 Mb, and CFA 24q11.1-q24:3.06–39.45 Mb and a partial gain at CFA 9q22.3:36.29–41.71 Mb. Data from oaCGH were supported by FISH analysis on all three lots of MDCK cells, the results of which are summarized in Fig. 7.

## Discussion

The CKB1-3T7 cell line offers a unique tool for the in vitro evaluation of spontaneous neoplastic activity. The generation of cell banks from CKB1-3T7 at regular intervals permits characterization of the progressive evolution of biological and genomic events involved in the pathway from initiation to immortalization and the acquisition of a tumorigenic phenotype. We propose that this series of well-characterized reagents also fills a critical niche in our ability to comprehend the fundamental requirements for expression of a tumorigenic phenotype in vivo. The CKB1-3T7 cell line exhibited several key biological properties that support its use in this context, including a gradual decrease in doubling time accompanied by an increase in growth rate (Fig. 1a), loss of contact inhibition (SOM Fig. 1C), and a gradual increase in tumorigenicity (Fig. 1b). In addition, wound-healing assays revealed a progressive increase in the cell motility of CKB1-3T7 cells over continued passage (SOM Fig. 2). We have shown a similar association between cell migration and tumor-forming ability in the VERO line of AGMK cells (Teferedegne et al. 2010, 2014).

Although key activities associated with the development of a neoplastic phenotype were exhibited by CKB1-3T7 cells, there was a deviation from the generally accepted order of events. For example, there was a lack of association between loss of contact inhibition and tumor formation in CKB1-3T7 cells. It is generally considered that cells need an unrestricted capacity to divide in order to form tumors in vivo. CKB1-3T7 cells, however, expressed the capacity to form tumors in newborn athymic nude mice at p51, and in both newborns and adults at p81, passages when their growth remained inhibited by cell-to-cell contact in culture. From p52 to p82, population DT decreased by 26 % from 42.7 to 31.7 h. At p90, when loss of contact inhibition occurred, the DT declined further only modestly to 28.1 h. We are not aware of prior studies that assessed the effects of DT and contact inhibition on the tumor-forming ability of cells immortalized during spontaneous neoplastic development in vitro. There are two possible explanations for the apparent lack of correlation between the loss of contact inhibition and the acquisition of a tumorigenic phenotype. First, while the presence of a small number of the 10^7^ cells comprising the inoculum is likely sufficient to form a tumor in vivo, loss of contact inhibition in vitro requires that most of the cells have acquired this property. Second, it is not known whether cells that are contact inhibited in vitro are also contact inhibited in vivo, where factors in the microenvironment might allow cells to overcome such inhibition.

Perhaps the most dramatic property exhibited by sequential passages of CKB1-3T7 cells was the progressive reduction in tumor latency, falling from 28 weeks (p51) to 2 weeks (p90) in newborn nude mice and from 53 weeks (p60) to 2 weeks (p90) in adult nude mice (Fig. 1b). From p62 to p92, the DT decreased from 30.4 to 28.1 h (Fig. 1a), which seems disproportionately small compared with the marked reduction in tumor latency, suggesting that these events may not be related directly. Understanding the relationship between DT, tumor incidence, tumor latency, and the loss of contact inhibition requires, in part, knowledge of the genomic alterations occurring in concert with these phenotypic changes. A limited number of studies have attempted to identify genomic alterations that correlate with the spontaneous emergence of immortalization and tumorigenicity in vitro. Certain discrete CNAs have been shown to be conserved between independent clones developed from a common parental cell line, suggestive of a nonrandom etiology [for example (Ray et al. 1986)], but no comprehensive genome-wide studies exist. Despite long-term propagation in culture, the majority of CNAs evident in CKB1-3T7 cells (as late as p92), and also in MDCK cells (beyond p55), were whole chromosome aneuploidies rather than focal imbalances of individual genes or discrete subchromosomal regions (Fig. 5). This suggests that the development of a tumorigenic phenotype may occur within a background of limited genomic instability, at least in terms of DNA copy number. Detailed characterization of structural, mutational, transcriptional, and epigenetic alterations in CKB1-3T7 cells will be required to reveal the relative contribution of these factors in the acquisition of a tumorigenic phenotype.

Among the small number of targeted genomic imbalances detected in CKB1-3T7 cells was focal deletion of a 1.04-Mb region at CFA 11q16:44.17–45.20 Mb, which encodes the *MTAP*, *CDKN2A*/*B*, and *DMRTA1* loci. Deletion of CFA 11q16:44.17–45.20 Mb was one of the earliest detectable CNAs, evident first at p15 as a hemizygous deletion in a proportion of the cell population, and increasing dramatically in penetrance over the next 30 passages before plateauing from p43 onwards as a homozygous deletion (Fig. 3). The progressive loss of this locus during sequential passage in vitro paralleled the progressive decline in the DT and an increase in growth rate of CKB1-3T7 cells (Figs. 1a and 2). The genomic boundaries of this deletion remained stable from initial onset at p15 to apparent homozygous deletion by p92, representing an interstitial deletion within the midregion of CFA 11, with the remainder of the chromosome remaining grossly intact. Each of the three MDCK lots also exhibited homozygous deletion of *CDKN2A*/*B*, spanning a markedly smaller interval of 60 kb at CFA 11q16:44.23–44.29 Mb that lay below the limits of detection by BAC-based SLP analysis. *CDKN2A*/*B* represents a cyclin-dependent kinase inhibitor that binds to cyclin-dependent kinases (CDK4, CDK6) and halts the cell cycle in the G_1_ phase, thus arresting cell proliferation. Focal deletions involving the *MTAP* and *CDKN2A*/*B* loci on CFA 11q16 are among the most highly recurrent somatic CNAs in a broad range of naturally occurring canine cancers, including appendicular osteosarcoma, histiocytic sarcoma, and non-Hodgkin’s lymphoma (Angstadt et al. 2011; Hedan et al. 2011; Thomas et al. 2011; Karlsson et al. 2013). This region also harbors germline risk factors for both osteosarcoma and histiocytic sarcoma in dogs (Shearin et al. 2012; Karlsson et al. 2013). This association with both somatic and germline aberrations in spontaneous canine cancer, together with the temporal relationship between copy number loss and tumor-forming capacity in both CKB1-3T7 cells and MDCK cells, emphasizes the fundamental role of this locus as a driver of neoplastic activity both in vivo and in vitro. Interstitial deletion of human chromosome 9p21, resulting in loss of *CDKN2A*/*B*, is also widely regarded to be a critical event in the development of numerous diverse human cancers (for example, Kohno and Yokota 2006; Schiffman et al. 2010; Sarhadi et al. 2013; LaPak and Burd 2014; Su et al. 2014). Furthermore, we (unpublished observations) and others (Osada et al. 2014) have recently identified the same aberration in both novel and established cell lines of the African green monkey. The conservation of this molecular event in three species not only points to the significance of these genes in cancer development but might also offer new opportunities to elucidate mechanisms of double-strand breakage and rejoining involved in tumorigenesis through a comparative structural analysis of chromatin architecture.

The onset of the focal deletion of CFA 11q16:44.17–45.20 Mb at p15 in CKB1-3T7 cells was accompanied by monosomies of CFA 27 and CFA 36. Aside from the progressively increasing amplitude of the CFA 11q16 deletion, and progressive loss of CFA 27, the overall genome-wide DNA copy number profiles of CKB1-3T7 cells remained stable during the interval between p15 and p32. By p43, several changes to the CNA profiles became evident, which were verified by targeted FISH analysis (Fig. 4). Interestingly, however, the single-copy loss of CFA 27 was no longer evident from p43 onwards. This return to a balanced state was suggestive of an in vitro form of uniparental disomy (UPD) through replication of the one remaining copy of CFA 27. It is tempting to consider that this “monosomy rescue” event may be causally related to the subsequent evolution of a neoplastic phenotype in CKB1-3T7 cells during propagation to p52 through the unmasking of cancer-associated mutations via copy number neutral loss of heterozygosity. The incidence of UPD and the relationship between UPD and cancer remain enigmatic. This is due in large part to the historical challenges associated with its detection, particularly where only a single snapshot of genomic status in vivo is available for evaluation. To our knowledge, there have been no descriptions of this phenomenon in the dog. The series of sequential passages of the CKB1-3T7 cell line thus offers an opportunity to monitor the onset and consequences of UPD in an in vitro system.

CKB1-3T7 cells exhibited a second transient CNA in the form of a targeted gain of 9.6 Mb at CFA 5q34-q35:76.21–85.77 Mb, which manifested as a duplication on one copy of CFA 5 (Fig. 4a). This aberration was evident in 9 % of cells at p62 and persisted in 13 % of cells through p73 before returning to a balanced state by p92 (Fig. 4a). The proximal boundary of this region lies within the canine ortholog of the *WWOX* gene. *WWOX* (WW domain containing oxidoreductase) spans one of the two most active fragile sites in the human genome (FRA16D, human chromosome 16q23) and is disrupted frequently in several human cancers (Drusco et al. 2011). There was no evidence for CNA surrounding the canine *FHIT* gene on CFA 20q14, which lies within the other highly active fragile site in the human genome. FISH analysis revealed that the frequency of the hemizygous duplication at CFA 5q34-q35 fell to a level that was undetectable by CGH analysis at p92. It is possible that this was due to the emergence of an abnormal subclone of CKB1-3T7 that was subsequently diluted out over continued culture. Alternatively, this may represent a second example of apparent UPD, in which the aberrant copy of CFA 5 present at p62 and p73 was lost during culture to p92, perhaps due to some deleterious effect, and the remaining, grossly normal CFA 5 duplicated to result in a return to balanced status. As with the transient loss of CFA 27, there is, however, no clear explanation for this observation.

Gain of CFA 13, which was first detected in CKB1-3T7 cells at p43, was also evident in MDCK V2 and V3 (Fig. 5). In common with deletion of *CDKN2A*/*B*, CFA 13 gain is among the most highly recurrent CNAs across the spectrum of spontaneously established tumor cell lines and naturally occurring canine cancers, including appendicular osteosarcoma, histiocytic sarcoma, glioma, non-Hodgkin’s lymphoma, mucosal melanoma, and urothelial carcinoma (Thomas et al. 2009, 2011; Angstadt et al. 2011; Hedan et al. 2011; Seiser et al. 2013; Poorman et al. 2015; Shapiro et al. 2015). Interestingly, CFA 13 gain was also among the first CNAs to evolve in a panel of induced pluripotent stem (iPS) cells generated from normal adult canine fibroblasts, which developed low-level aneuploidy during prolonged culture in conjunction with the ability to produce solid tumors in mice (Koh et al. 2013). The CFA 13 gain therefore appears to be an early event in the genomic evolution of spontaneous tumorigenesis both in vitro and in vivo. CFA 13 harbors several key cancer genes, most notably the c-*MYC* and c-*KIT* proto-oncogenes; however, CFA 13 gain manifests primarily as a whole chromosome aneuploidy, in contrast to the recurrent focal deletion of CFA 11q16 (Thomas et al. 2009, 2011; Angstadt et al. 2011; Hedan et al. 2011; Seiser et al. 2013; Poorman et al. 2015; Shapiro et al. 2015). In combination, this suggests that CFA 13 gain may be a secondary event that is more closely related to the architecture of the canine karyotype rather than being fundamental to the onset of a tumorigenic phenotype. The same may apply to loss of CFA 16, which, like CFA 13 gain, was first detected in CKB1-3T7 cells at p43. In common with CFA 13 gain, loss of CFA 16 is also common to several spontaneous canine cancers, particularly soft tissue sarcomas, including appendicular osteosarcoma, hemangiosarcoma, histiocytic sarcoma, and non-Hodgkin’s lymphoma (Angstadt et al. 2011; Hedan et al. 2011; Thomas et al. 2011, 2014; Karlsson et al. 2013). This CNA was not, however, detected in any of the MDCK variants. Conversely, the CKB1-3T7 cell line and all three MDCK variants accumulated gain of CFA 14 (Fig. 5). This aberration became evident in CKB1-3T7 cells only at p92, several passages after the acquisition of a tumorigenic phenotype and once cells had lost contact inhibition and tumor latency fell to 2–3 weeks (SOM Fig. 1B, C). Gain of CFA 14 may therefore represent a secondary event associated with prolonged culture and increasingly dysregulated cell proliferation. This may apply equally to those additional CNAs (gain of CFA 1, CFA 9, and CFA 38 and loss of CFA 18) that occurred in CKB1-3T7 cells only after prolonged culture beyond p62 (Fig. 2).

Despite the extensive use of MDCK cells as a research tool over the past five decades, to our knowledge, this report represents the first genome-wide molecular cytogenetic characterization of this cell line. Our findings concur with prior reports (Cassio 2013) indicating that the total chromosome number and morphology of MDCK cells has remained close to that of the normal dog karyotype (2*n* = 78), with acrocentric autosomes and submetacentric sex chromosomes (Breen et al. 1999a). Our findings do, however, contradict prior reports suggesting the presence of a Y chromosome and/or origin from a male donor [for example, (Gaush et al. 1966; Noma et al. 1998)]. These contrasting observations in part reflect the challenge of accurate dog chromosome identification using conventional banding analysis. It is, however, becoming increasingly apparent that inconsistencies between studies involving MDCK cells may arise due to the use of different variants of this cell line. Several derivatives of the original MDCK line are in use today, with extensive heterogeneity evident in their cellular morphology and biological behavior (Dukes et al. 2011; Omeir et al. 2011). Furthermore, MDCK cells exhibit additional genomic and phenotypic “drift” during continued propagation (Dukes et al. 2011; Cassio 2013). Provenance is a particular concern when cell lines are sourced from individual laboratories rather than from centralized repositories, and resulting data frequently are reported without detailed description of their origin, culturing conditions, and number of population doublings since initiation. The present study offers a panel of widely accessible and biologically characterized reagents that can serve as a baseline reference for standardization and integration of future studies utilizing the CKB1-3T7 cell line.

The mechanistic association between genomic instability and cancer in humans has been explored in depth (Thompson and Compton 2011), and it is increasingly evident that seemingly limited defects can have substantial consequences. For example, the renowned Philadelphia chromosome rearrangement in chronic myelogenous leukemia is sufficient to drive the disease (Chandra et al. 2011; Frankfurt and Licht 2013), and there is evidence that artificial induction of a single chromosome aberration may drive the onset of in vitro neoplastic transformation in vertebrate cells (Gascoigne and Cheeseman 2013). Our understanding of these dynamic processes remains incomplete, however, particularly in the definition of cause versus effect and the temporal basis of these events. We have shown previously that chromosome aberrations in naturally occurring canine cancers parallel those of their human counterparts (Breen and Modiano 2008; Thomas et al. 2009, 2011, 2014; Angstadt et al. 2011; Karlsson et al. 2013; Poorman et al. 2015; Shapiro et al. 2015) and also coincide with genomic rearrangements that arise during speciation (Becker et al. 2011). The present study highlights a subset of chromosome aberrations that are also common to spontaneous neoplastic development in vitro and reveals their temporal relationships in context with the evolution of biological behavior. Continued assessment of genomic characteristics that are shared between in vitro models and naturally occurring disease across genetically distinct species should aid in the elucidation of driving factors that are fundamentally involved in the onset of these phenotypes. The CKB1-3T7 cell line offers a systematic tool for evaluating safety concerns associated with vaccines developed in immortalized cells and for identification of novel targets for effective modulation of tumorigenesis in spontaneous cancers.

## Electronic supplementary material

SOM Fig. 1 Morphological evolution of CKB1-3T7 cell monolayers assessed at p15 (A), p50 (B) and p90 (C). Phase-contrast micrographs were taken at 10X using a Nikon Diaphot camera (p15) and at 4X using an Olympus 1X51 microscope with a DP72 camera (p50 and p90). (PDF 10069 kb)

SOM Fig. 2 The migratory capacity (*i.e*., the capacity to heal a wound created by scratching a confluent monolayer) of CKB1-3T7 cells at p32, p42, p52, p74, and p94 over 36 h is shown. (PDF 996 kb)

SOM Fig. 3 Overview of CNAs identified by genome-wide oaCGH analysis of the CKB1-3T7 cell line during extended culture. Each image summarizes the distribution and amplitude of CNAs identified at one of nine timepoints (passages), indicated at the top of the image. The DNA copy number status along the length of each chromosome, relative to the reference, is shown beside the dog DAPI-banded ideogram set (Breen et al. 1999a). Genomic imbalances, defined as regions with log_2_ test:reference values ≥0.2 (copy number gain) or ≤−0.2 (copy number loss), are denoted by blue and red shading of the ideogram for that chromosome. Boxes of the same color highlight key CNAs emerging during the progression of the CKB1-3T7 cell line (see text for discussion). (PDF 1568 kb)

SOM Table 1 Clones from the CHORI-82 canine BAC library (https://bacpac.chori.org/library.php?id=253) used to generate single-locus FISH probes as part of the current study. Panels 3T7 i and ii were used to detect and quantify copy number status in cells from progressive passages of the cell line 3T7. Panels MDCK i and ii were used to detect and quantify copy number status in three lots of MDCK cells obtained from ATCC. (PDF 287 kb)

## Abbreviations

AGMK: African green monkey kidney
ATCC: American Type Culture Collection
BAC: Bacterial artificial chromosome
CFA: *Canis familiaris*
CNA: Copy number aberration(s)
CDKN: Cyclin-dependent kinase inhibitor
CHORI: Children’s Hospital Oakland Research Institute
DMEM: Dulbecco’s modified Eagle’s medium
DT: Doubling time
FBS: Fetal bovine serum
FISH: Fluorescence in situ hybridization
IACUC: Institutional Animal Care and Use Committee
iPS cells: Induced pluripotent stem cells
MDCK: Madin-Darby canine kidney
NIH: National Institutes of Health
oaCGH: Oligonucleotide-array comparative genomic hybridization
p: Passage
PAR: Pseudoautosomal region
PBS: Phosphate-buffered saline
PDL: Population doubling level
RPCI: Roswell Park Cancer Institute
SLP: Single-locus probe
UCSC: University of California Santa Cruz
UPD: Uniparental disomy
