# Supplementary figures and images for: A novel canine kidney cell line model for the evaluation of neoplastic development: karyotype evolution associated with spontaneous immortalization and tumorigenicity
Source: Chromosome Res. 2015 May 10;23(4):663–80. doi: 10.1007/s10577-015-9474-8 (PMC4666904; doi:10.1007/s10577-015-9474-8)

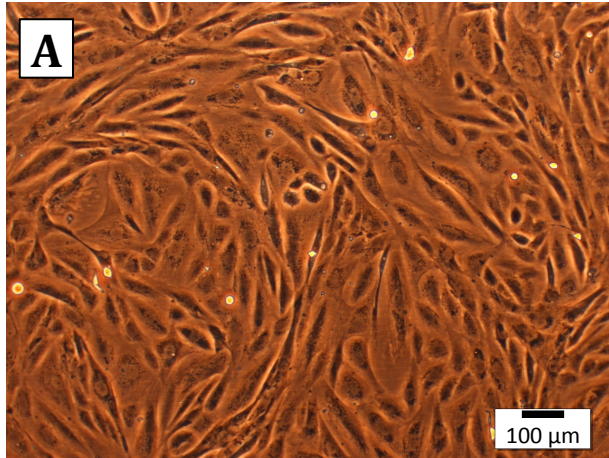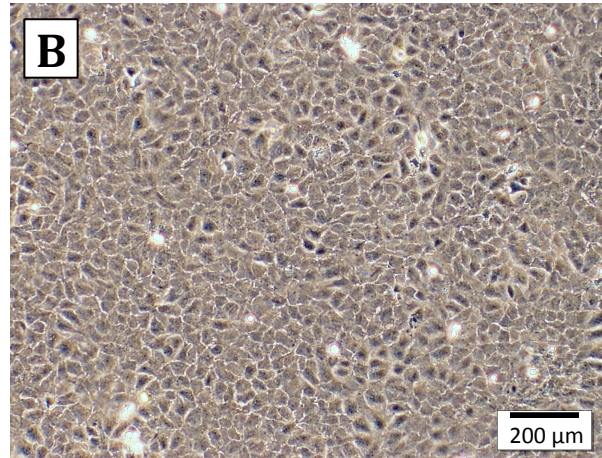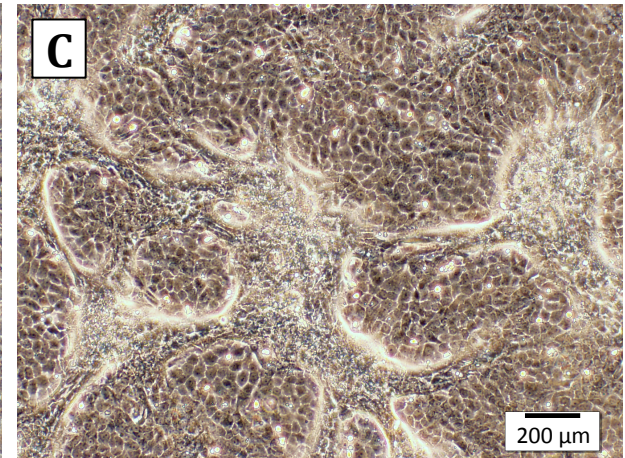

SOM Figure 1

Supplement: Supplementary file 1 — Morphological evolution of CKB1-3T7 cell monolayers assessed at p15 (A), p50 (B) and p90 (C). Phase-contrast micrographs were taken at 10X using a Nikon Diaphot camera (p15) and at 4X using an Olympus 1X51 microscope with a DP72 camera (p50 and p90). (PDF 10069 kb) [file 10577_2015_9474_MOESM1_ESM.pdf]

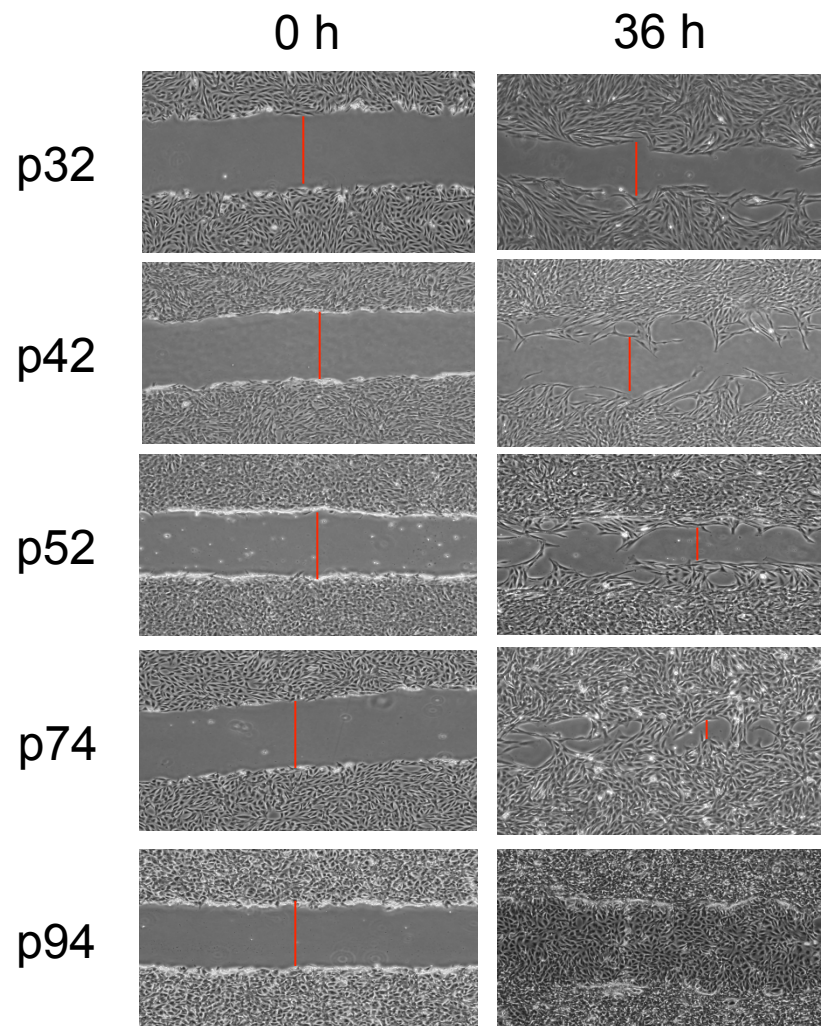

Supplement: Supplementary file 2 — The migratory capacity (i.e., the capacity to heal a wound created by scratching a confluent monolayer) of CKB1-3T7 cells at p32, p42, p52, p74, and p94 over 36 h is shown. (PDF 996 kb) [file 10577_2015_9474_MOESM2_ESM.pdf]

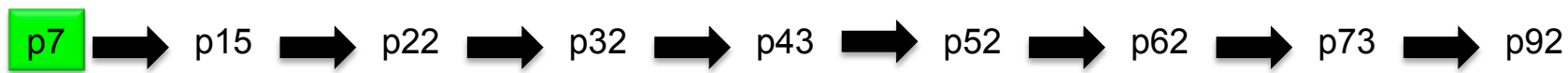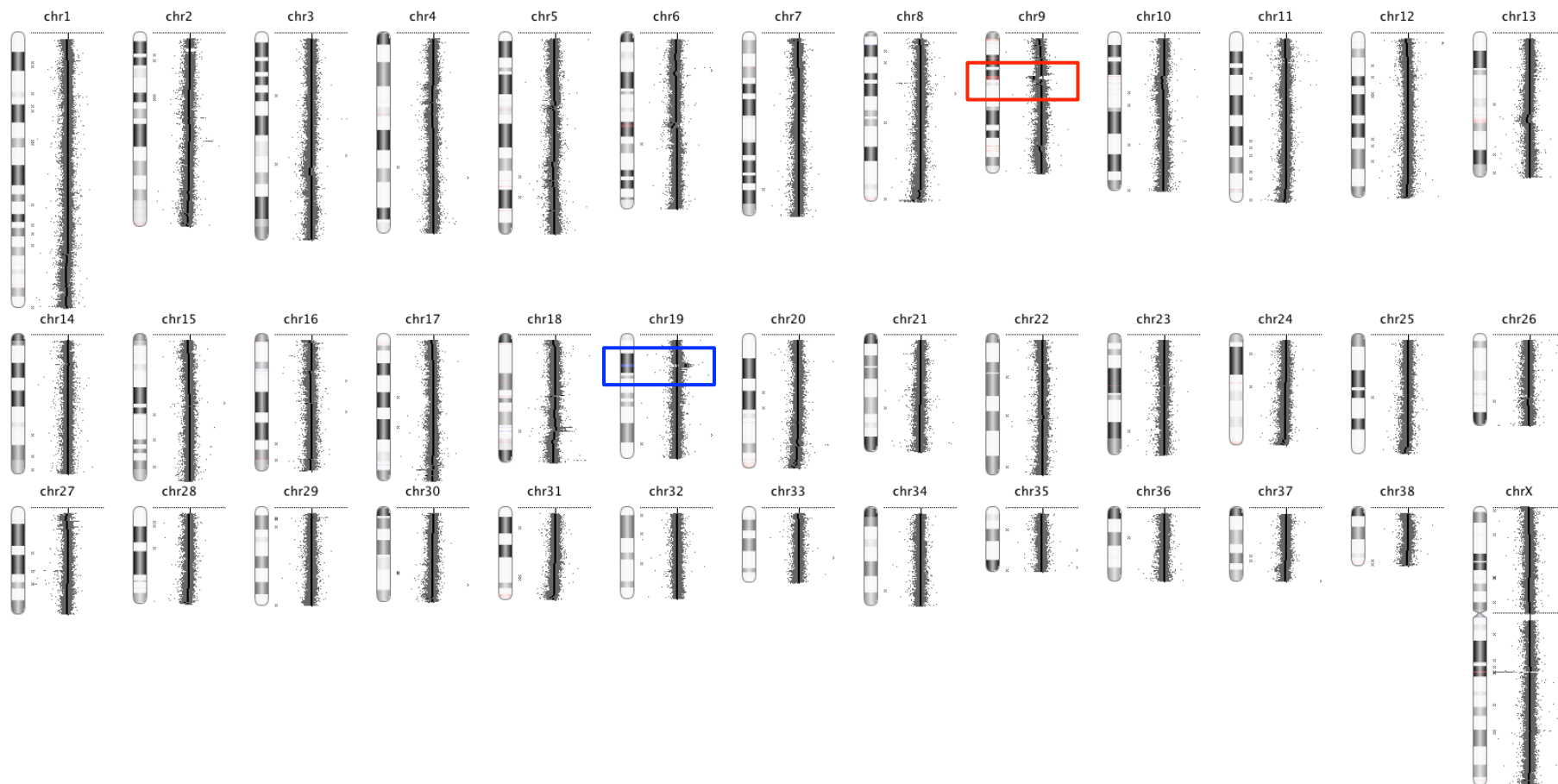

p7 → **p15** → p22 → p32 → p43 → p52 → p62 → p73 → p92

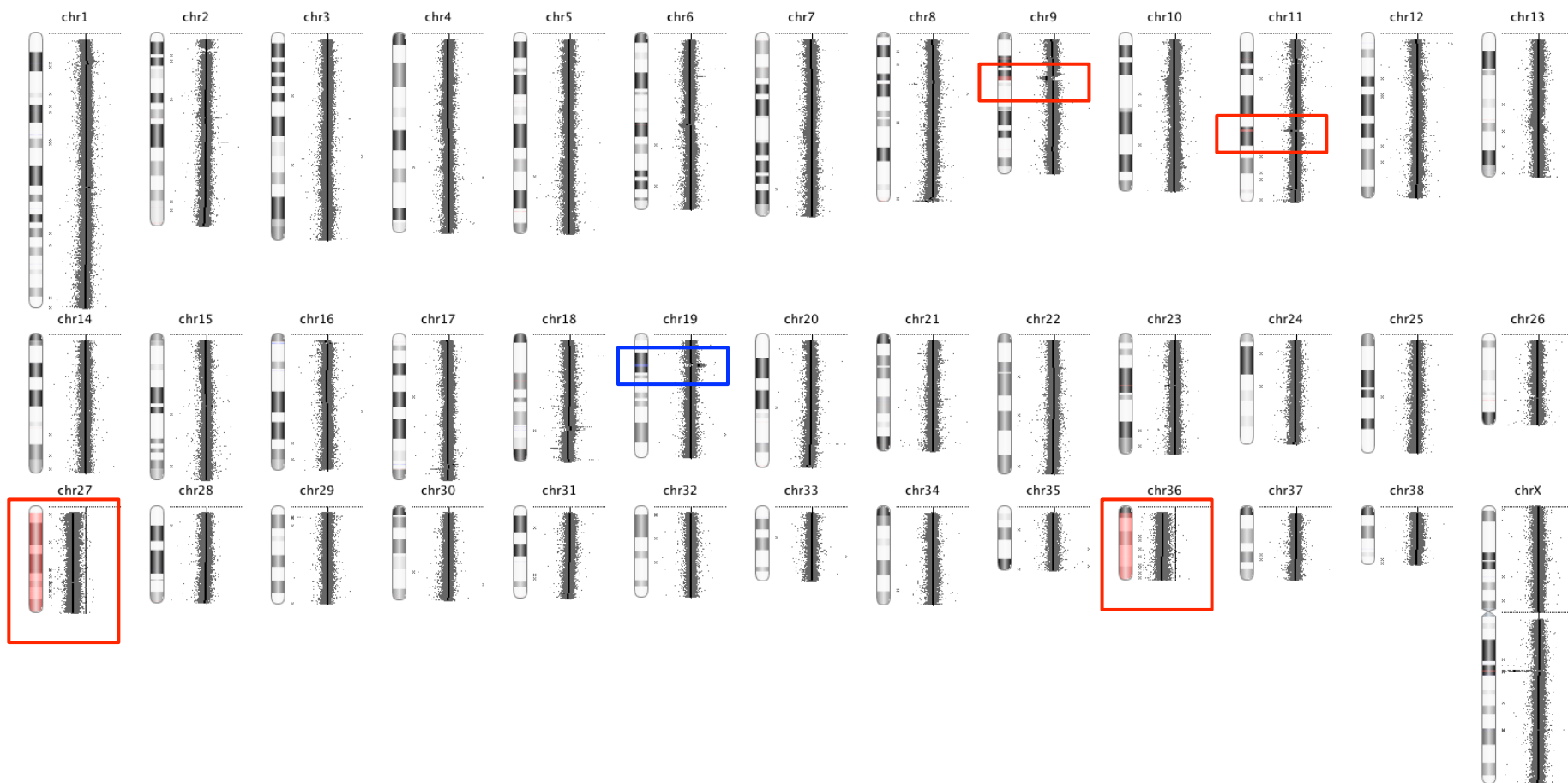

p7 → p15 → **p22** → p32 → p43 → p52 → p62 → p73 → p92

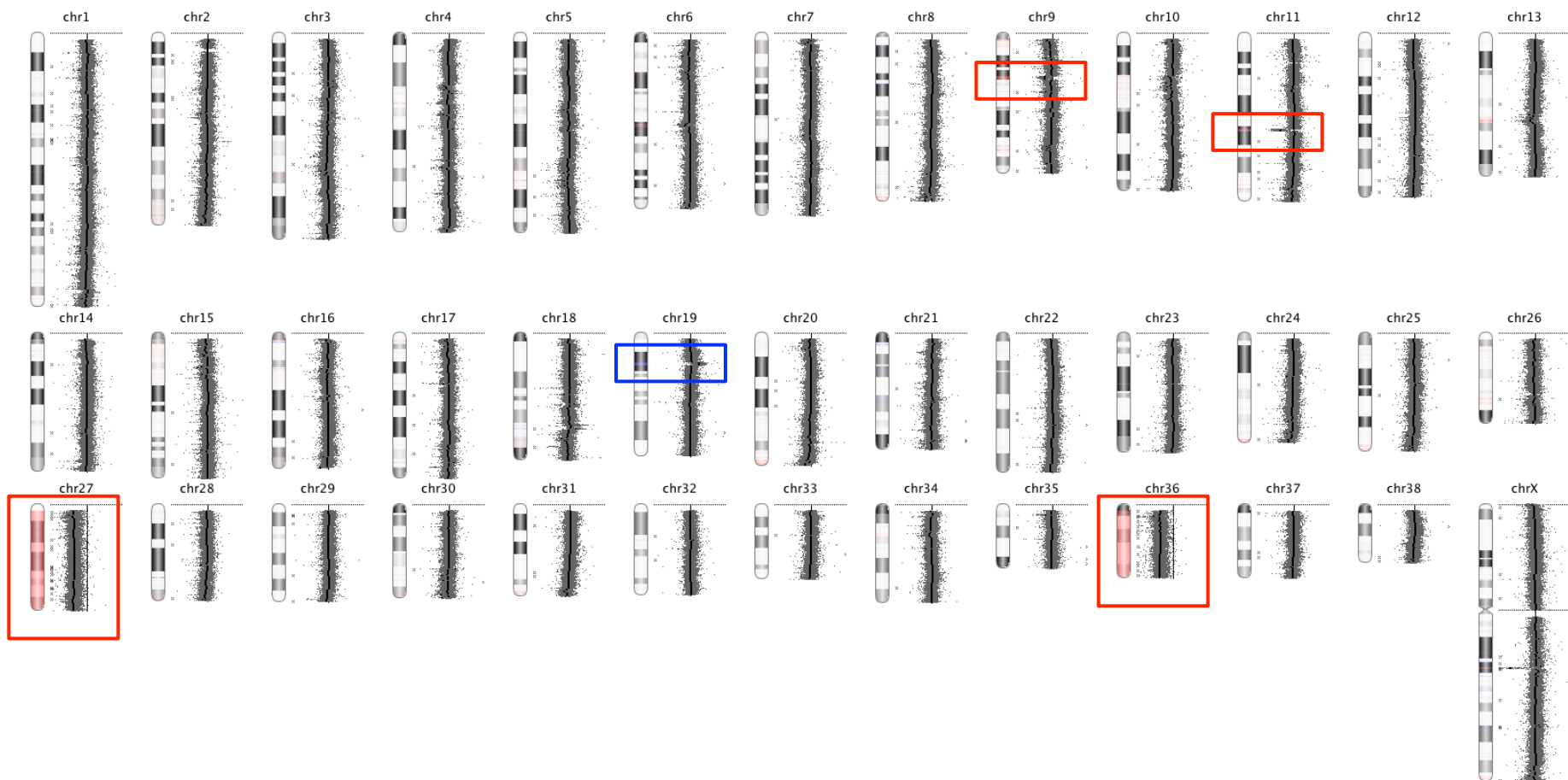

p7 → p15 → p22 → **p32** → p43 → p52 → p62 → p73 → p92

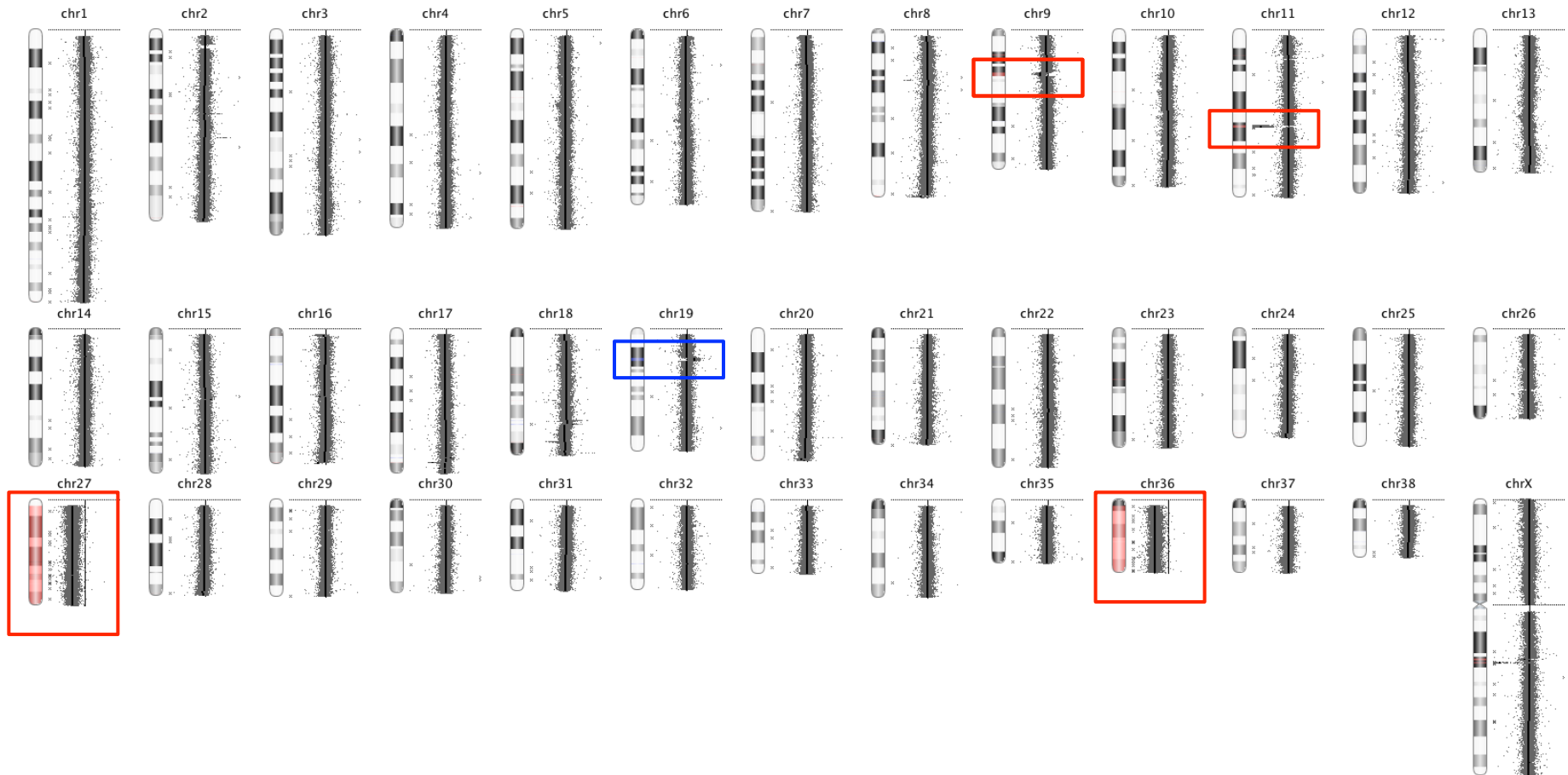

p43

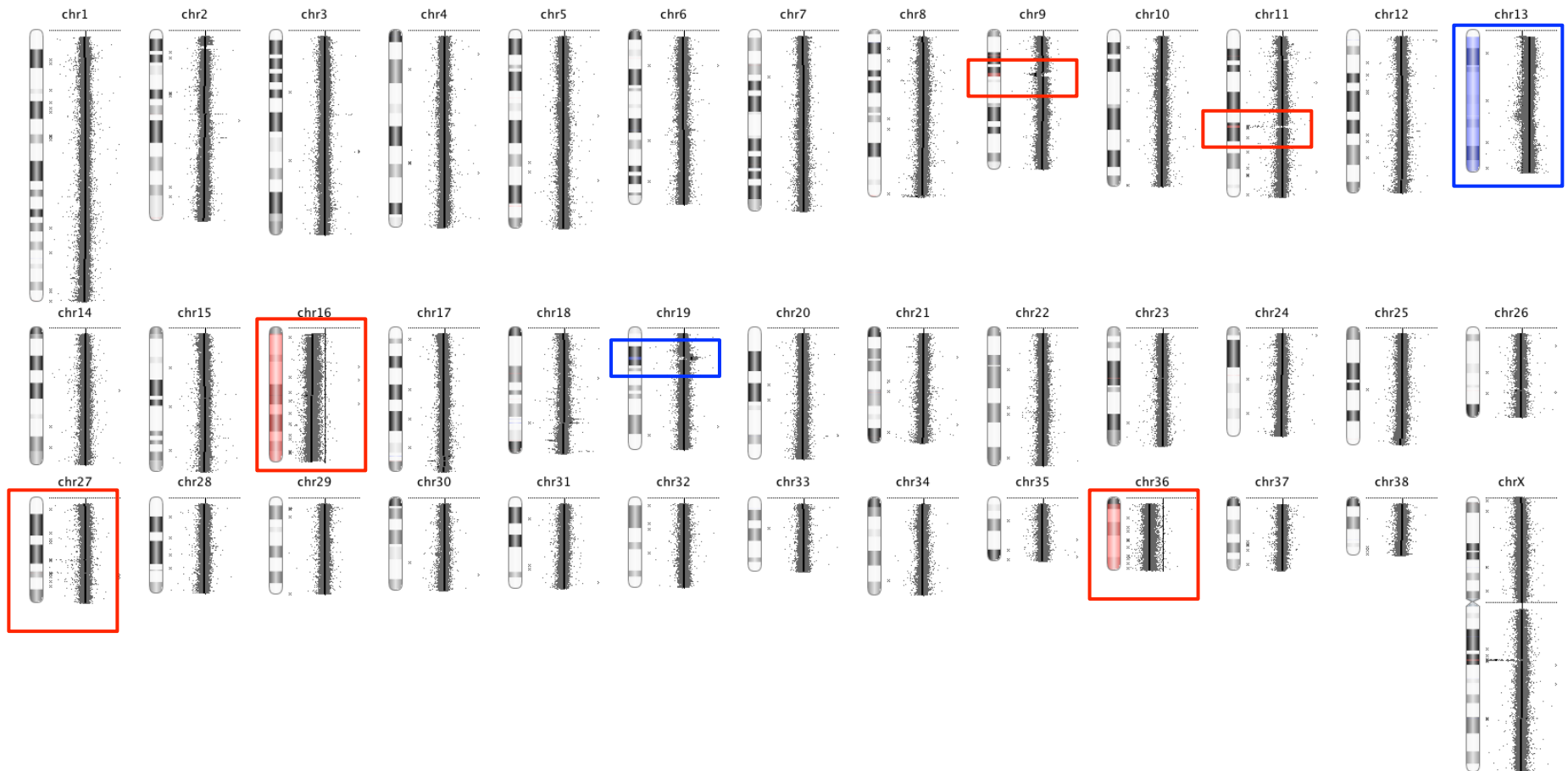

p7 → p15 → p22 → p32 → p43 → p52 → p62 → p73 → p92

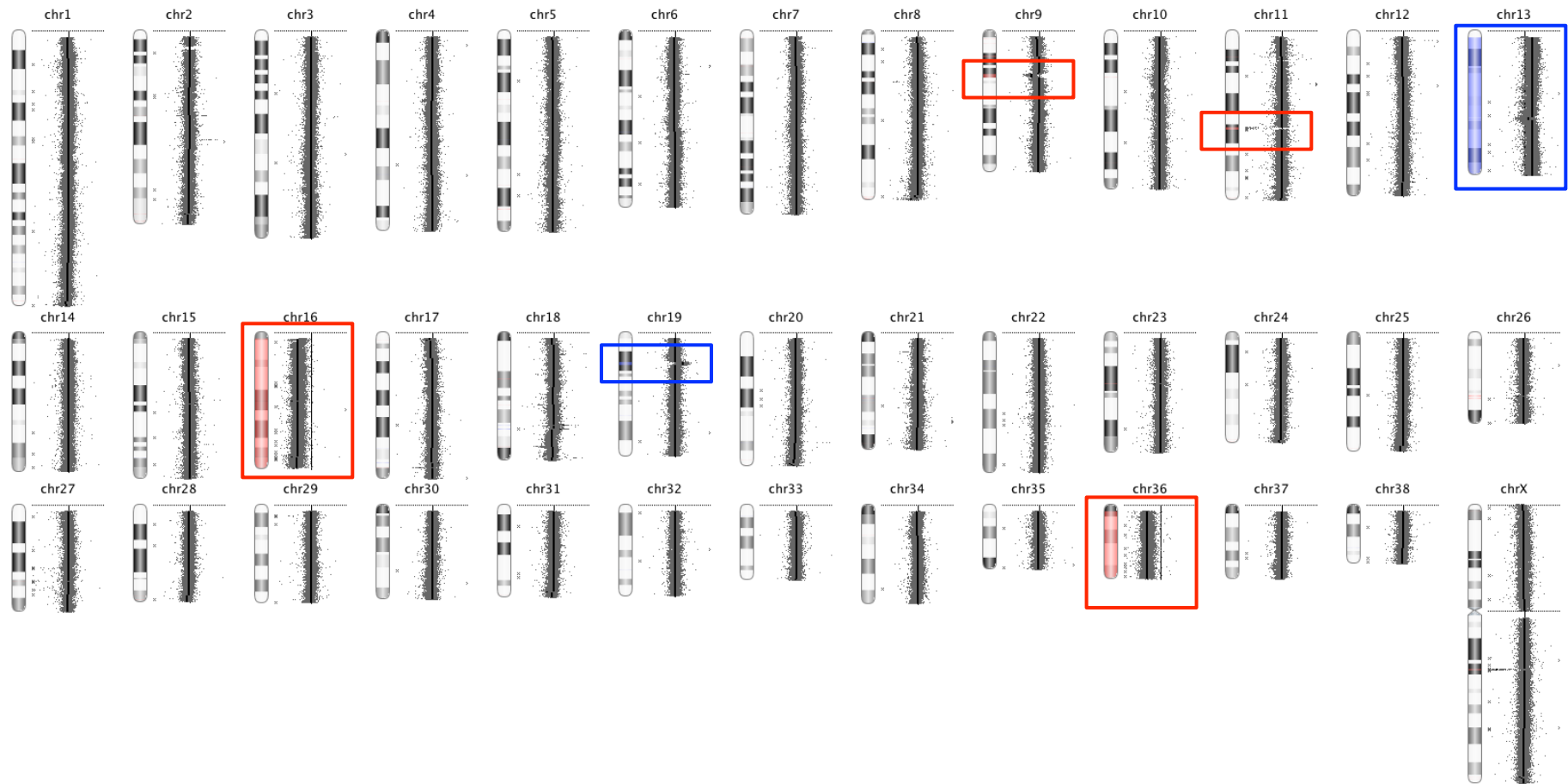

p7 → p15 → p22 → p32 → p43 → p52 → p62 → p73 → p92

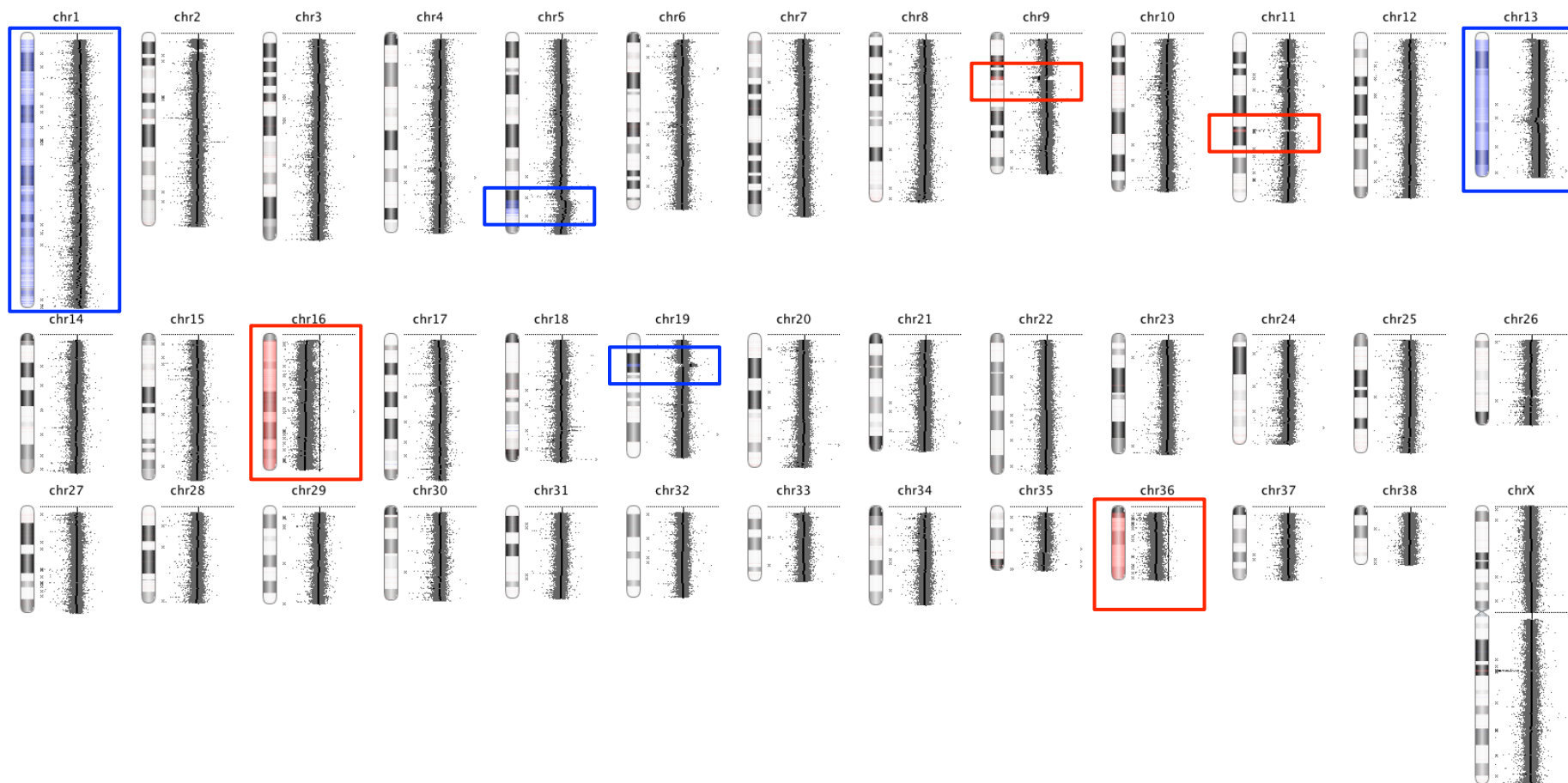

p7 → p15 → p22 → p32 → p43 → p52 → p62 → **p73** → p92

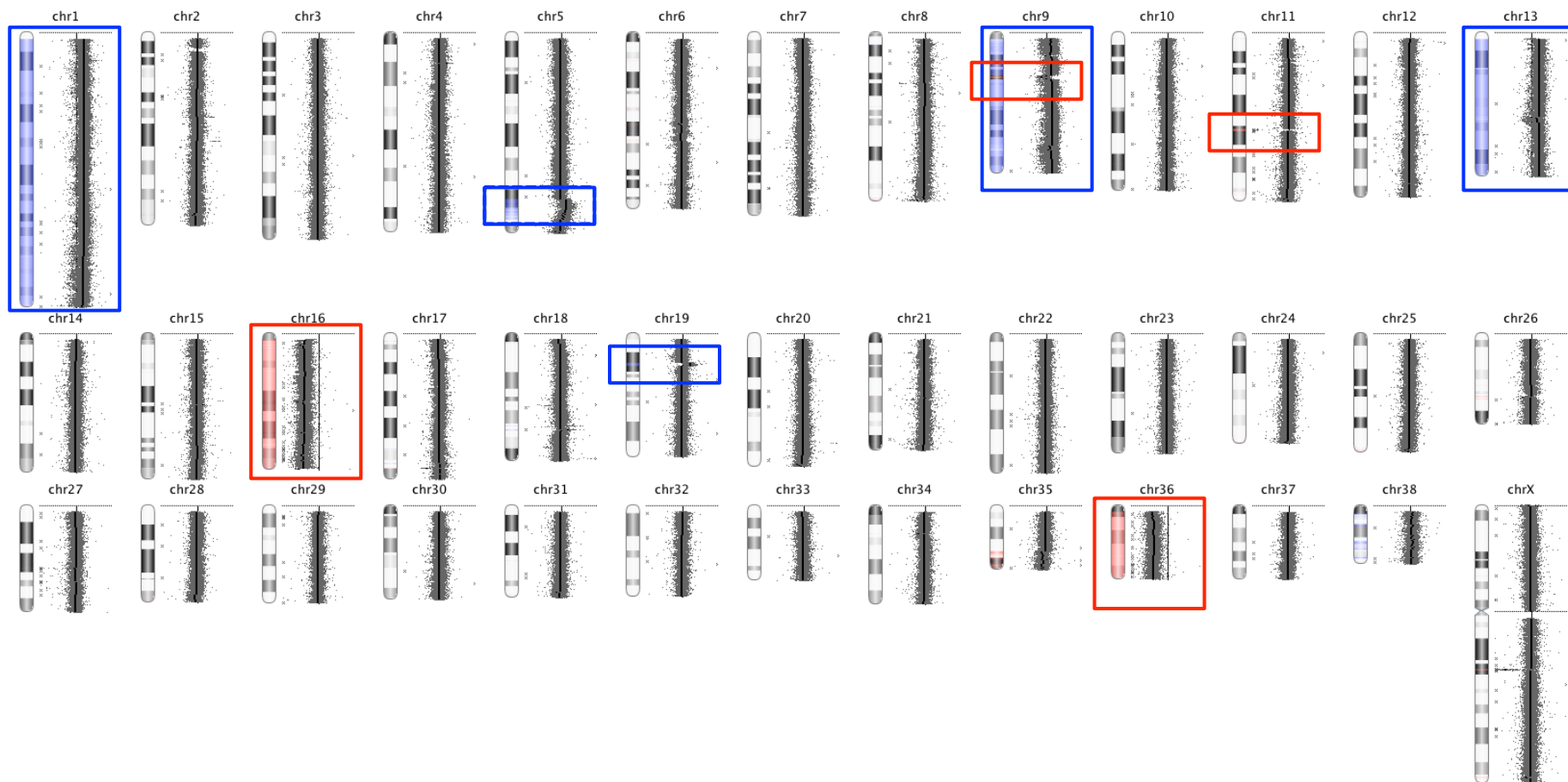

p7 → p15 → p22 → p32 → p43 → p52 → p62 → p73 → p92

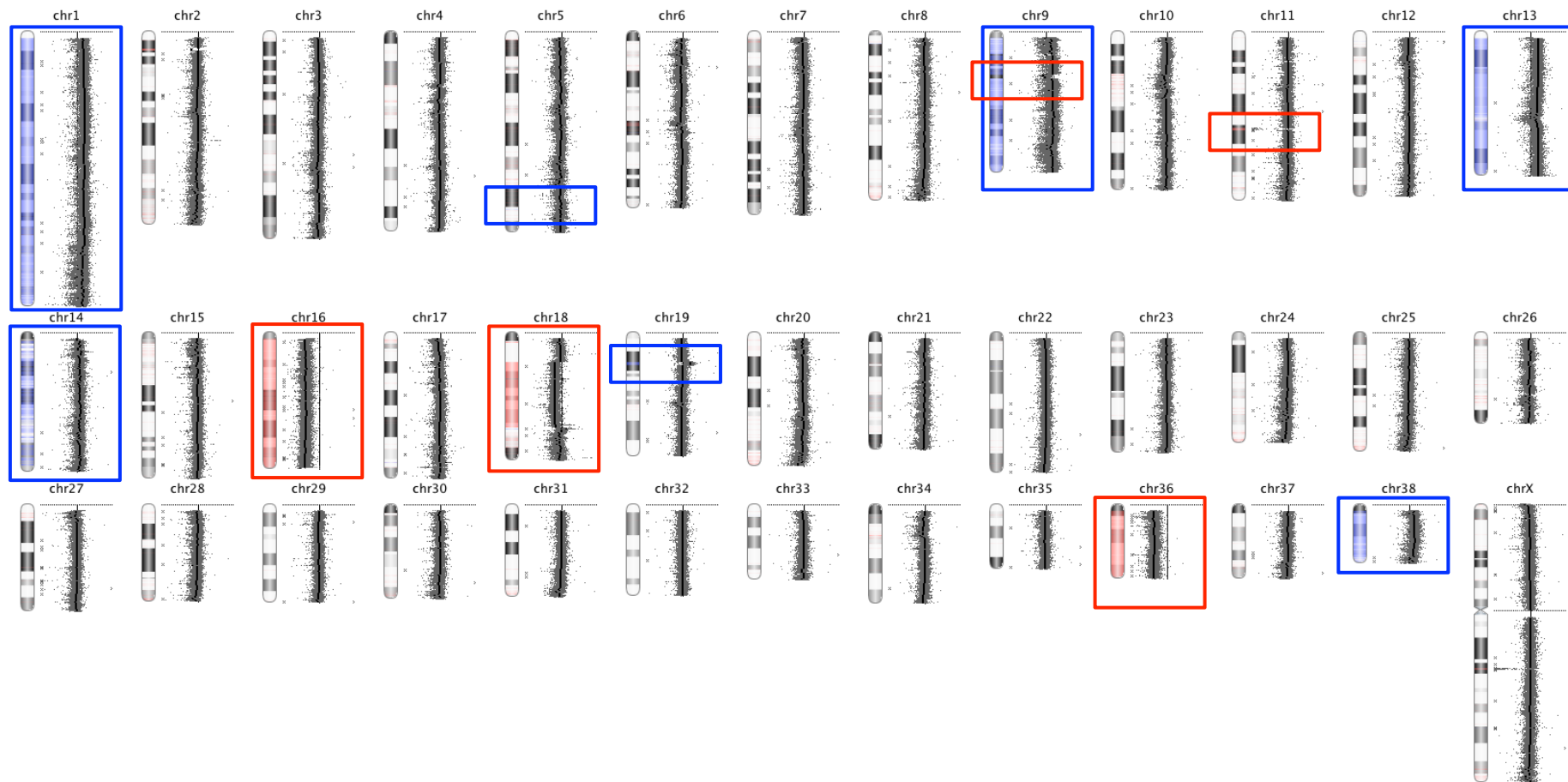

Supplement: Supplementary file 3 — Overview of CNAs identified by genome-wide oaCGH analysis of the CKB1-3T7 cell line during extended culture. Each image summarizes the distribution and amplitude of CNAs identified at one of nine timepoints (passages), indicated at the top of the image. The DNA copy number status along the length of each chromosome, relative to the reference, is shown beside the dog DAPI-banded ideogram set (Breen et al. 1999a). Genomic imbalances, defined as regions with log2 test:reference values ≥0.2 (copy number gain) or ≤−0.2 (copy number loss), are denoted by blue and red shading of the ideogram for that chromosome. Boxes of the same color highlight key CNAs emerging during the progression of the CKB1-3T7 cell line (see text for discussion). (PDF 1568 kb) [file 10577_2015_9474_MOESM3_ESM.pdf]
