# Supplementary material for: A novel canine kidney cell line model for the evaluation of neoplastic development: karyotype evolution associated with spontaneous immortalization and tumorigenicity
Source: Chromosome Res. 2015 May 10;23(4):663–80. doi: 10.1007/s10577-015-9474-8 (PMC4666904; doi:10.1007/s10577-015-9474-8)
Supplement: Supplementary file 4 — Clones from the CHORI-82 canine BAC library (https://bacpac.chori.org/library.php?id=253) used to generate single-locus FISH probes as part of the current study. Panels 3T7 i and ii were used to detect and quantify copy number status in cells from progressive passages of the cell line 3T7. Panels MDCK i and ii were used to detect and quantify copy number status in three lots of MDCK cells obtained from ATCC. (PDF 287 kb) [file 10577_2015_9474_MOESM4_ESM.pdf]

SOM Table 1: Clones from the CHORI-82 canine BAC library (https://bacpac.chori.org/library.php?id=253) used to generate single locus FISH probes as part of the current study. Panels 3T7 i (left) and ii were used to detect and quantify copy number in cells from progressive passages of the cell line 3T7. Panels MDCK i and ii were used to detect and quantify copy number in three lots of ATTC provided MDCK cells.

3T7 I (left) and ii (right)

BAC clone	Chr	Start (bp)	End (bp)
CH82-126D16	1	61,574,407	61,769,058
CH82-126M02	9	20,181,801	20,374,224
CH82-188L03	9	33,496,452	33,534,284
CH82-283D13	19	14,684,022	14,864,725
CFA27 paint/ CH82-283D01	27	21,603,805	21,801,550

BAC clone	Chr	Start (bp)	End (bp)
CH82-197C19	5	83,009,765	83,229,357
CH82-126M06	11	43,772,134	43,825,542
CH82-325C12	11	44,256,300	44,428,212
CH82-335M01	13	28,085,281	28,265,714
CH82-191G07	16	33,138,014	33,325,899

MDCK i (left) and ii (right)

BAC clone	Chr	Start (bp)	End (bp)
CH82-258G12	11	44,046,502	44,256,191
CH82-325C12	11	44,256,301	44,428,212
CH82-399C04	11	44,276,367	44,436,005
CH82-287P12	21	44,538,774	44,726,658
CH82-188B22	24	39,041,432	39,188,926

BAC clone	Chr	Start (bp)	End (bp)
CH82-185N21	8	32,919,284	33,056,872
CH82-191A04	13	33,701,934	33,908,969
CH82-126H12	14	33,096,787	33,271,891
CH82-186I14	17	33,672,640	33,854,434
CH82-313F14	38	11,977,642	12,180,890
